# An integrative systems biology approach to overcome venetoclax resistance in acute myeloid leukemia

**DOI:** 10.1371/journal.pcbi.1010439

**Published:** 2022-09-13

**Authors:** Michelle Przedborski, David Sharon, Severine Cathelin, Steven Chan, Mohammad Kohandel

**Affiliations:** 1 Department of Applied Mathematics, University of Waterloo, Waterloo, Ontario, Canada; 2 Princess Margaret Cancer Centre, Toronto, Ontario, Canada; University of Southern California, UNITED STATES

## Abstract

The over-expression of the Bcl-2 protein is a common feature of many solid cancers and hematological malignancies, and it is typically associated with poor prognosis and resistance to chemotherapy. Bcl-2-specific inhibitors, such as venetoclax, have recently been approved for the treatment of chronic lymphocytic leukemia and small lymphocytic lymphoma, and they are showing promise in clinical trials as a targeted therapy for patients with relapsed or refractory acute myeloid leukemia (AML). However, successful treatment of AML with Bcl-2-specific inhibitors is often followed by the rapid development of drug resistance. An emerging paradigm for overcoming drug resistance in cancer treatment is through the targeting of mitochondrial energetics and metabolism. In AML in particular, it was recently observed that inhibition of mitochondrial translation via administration of the antibiotic tedizolid significantly affects mitochondrial bioenergetics, activating the integrated stress response (ISR) and subsequently sensitizing drug-resistant AML cells to venetoclax. Here we develop an integrative systems biology approach to acquire a deeper understanding of the molecular mechanisms behind this process, and in particular, of the specific role of the ISR in the commitment of cells to apoptosis. Our multi-scale mathematical model couples the ISR to the intrinsic apoptosis pathway in venetoclax-resistant AML cells, includes the metabolic effects of treatment, and integrates RNA, protein level, and cellular viability data. Using the mathematical model, we identify the dominant mechanisms by which ISR activation helps to overcome venetoclax resistance, and we study the temporal sequencing of combination treatment to determine the most efficient and robust combination treatment protocol.

## Author summary

In this work, we develop a multi-scale systems biology approach to study the mechanisms by which the integrated stress response (ISR) activation helps to overcome venetoclax resistance in acute myeloid leukemia (AML). The multi-scale model enables the integration of RNA-level, protein-level, and cellular viability and proliferation data. The model developed in this work can predict several important features of the resistant AML cell lines that are consistent with experimental data. Further, our integrative systems biology approach led to the determination of the optimal combination treatment protocol.

## 1 Introduction

Leukemia is a group of hematological malignancies that is characterized by the uncontrolled production of abnormal blood cells that resist cell death. Research over the last few decades has drastically advanced our understanding of the genetic landscape of leukemia, enabling the development of targeted treatment options that have significantly improved patient prognosis. Since 1992, the overall five-year survival rate for leukemia increased by 15 percentage points to 59% [1]. However, despite these encouraging improvements, the survival rates for leukemia overall are still well below those of several solid cancers, such as prostate (81%), melanoma (79%), thyroid (95%), and breast (80%) [1].

There are several different types of leukemia, classified broadly based on the cell lineage (lymphoid or myeloid) and the rate of onset and progression of the disease (acute or chronic). For chronic lymphocytic leukemia (CLL), the five-year survival rate is 83% (up 14 percentage points since 1992), while for chronic myeloid leukemia (CML) it is 60% (up 24 percentage points since 1992), and for acute lymphocytic leukemia (ALL) it is 51% (up 24 percentage points since 1992). In comparison, the five-year survival rate for acute myeloid leukemia (AML) has not significantly improved in recent decades, and currently it stands at only 21% in adults and 64% in children [2]. These statistics highlight the complexity and aggressiveness of the disease; thus, improving the prognosis for AML patients will require potent and more targeted treatment strategies.

One such promising therapy [3], which has recently been approved as a standard treatment option for CLL and small lymphocytic lymphoma, is venetoclax (ABT-199), which is a Bcl-2-specific protein inhibitor. Bcl-2 is a pro-survival protein that blocks the mitochondrial apoptosis pathway by sequestering the pro-apoptosis proteins (Bim, Bax, Bak, etc.) and inhibiting their function. The over-expression of Bcl-2 is a common feature of many solid and hematological cancers [4, 5], and in AML it is associated with poor prognosis and patient relapse [6–8]. By binding to Bcl-2, venetoclax inhibits the effects of Bcl-2 over-expression and sensitizes the malignant cells to apoptosis. However, venetoclax monotherapy for leukemia is often followed by the development of drug resistance, typically over a period of months. The development of venetoclax resistance is facilitated by the up-regulation of other pro-survival proteins, such as Mcl-1 or Bcl-XL, which play roles in the apoptosis pathway that are redundant to Bcl-2 [9, 10].

Apart from the high incidence of relapse, drug resistance is the main contributor to the poor prognosis of AML [11]. The development of drug resistance in AML is a tremendously complicated process involving several mechanisms, which include, for example, the up-regulation of multi-drug resistance proteins and NF-kB (which activates the PI3K/AKT/mTOR pathway), FLT3 mutations, RAS mutations (which activate the PI3K/AKT/mTOR and Raf/MEK/ERK pathways), Bcl-2 mutations, and c-Myc mutations, and modifications to pathways that regulate reactive oxygen species (ROS) signalling [11]. While it has been demonstrated [12–16] that co-treatment with venetoclax and a selective inhibitor of another redundant pro-survival protein (such as Mcl-1) can overcome resistance to Bcl-2 inhibition in AML, both Mcl-1 [17–20] and Bcl-XL [21–23] are vital for the survival of normal blood cells. Hence their inhibition is profoundly toxic to healthy tissue, especially in combination with Bcl-2 inhibition. Consequently, to effectively overcome venetoclax resistance, more sophisticated methods that target only the leukemia cells (LCs) are required.

In addition to its role in apoptosis, recent work has demonstrated novel roles for Bcl-2 in modulating mitochondrial bio-energetics and in cancer metastasis and invasion [4]. Interestingly, mitochondrial energetics is emerging as a novel target for drug resistance in cancer [24], and it has been known for nearly a decade that inhibition of mitochondrial translation is a potent therapeutic target in human AML [25]. Recent work [26] has demonstrated that inhibiting mitochondrial translation via antibiotic administration, such as tedizolid, induces an integrated cellular stress response. The activation of the integrated stress response (ISR) was shown to be sufficient for re-sensitizing venetoclax-resistant AML cells to Bcl-2 inhibition, hence promoting apoptosis when administered in combination with venetoclax treatment in vitro and in vivo [26]. Importantly, the combination treatment was shown to target leukemic stem cells, which are known to promote patient relapse, with no overt evidence of toxicity [26]. While this combination treatment has shown promise for significantly improving patient outlook, many questions remain unresolved. For example, it is not clear what is the specific role of the ISR in the commitment of the LCs to apoptosis or what the time scale of the decision-making process is. Answering these questions can help to optimize the effectiveness of the combination treatment, including the optimal timing, order of administration, and dosing schedule.

Given the complexity of the processes involved in the survival and development of drug resistance in LCs, computational and mathematical modeling have been crucial to the understanding of leukemias and the improvement of patient prognosis [27]. The majority of mathematical models that have been developed for acute leukemias are simplistic compartmental cellular-level models to study leukemogenesis and cellular population dynamics and differentiation [28–39]. Other simplistic compartmental models have focused on treatment dynamics [40–47] and predicting patient relapse risk and outcome [48–53]. We direct the reader to Ref. [54] for a review of models specific to acute leukemias and Ref. [27] for a more general review of mathematical models of leukemia and lymphoma. Recently, a large-scale boolean network model of AML was developed [55]; however, very little work has gone into the development of quantitative molecular-level models of AML [56], and work in this direction has thus far focused only on developing simplistic, small-scale models of the PI3K/AKT pathway in AML [57, 58]. Here we address a crucial gap in the literature by developing a comprehensive molecular-level model to study venetoclax-resistance in AML.

Specifically, we develop a multi-scale systems biology approach that couples the ISR to the intrinsic apoptosis pathway in venetoclax-resistant AML cells and includes the metabolic effects of key proteins in cellular proliferation. Using the mathematical model, we simulate the effects of combination venetoclax-tedizolid therapy on the coupled pathway and calibrate the model using multi-modal experimental data. We perform sensitivity analysis to elucidate the main regulatory processes and simulate the system under different initial conditions and treatment schedules to determine the most efficient combination treatment protocol. This work leads to several key findings, including the identification of the dominant mechanisms by which ISR activation helps to overcome venetoclax resistance and the time scale of these effects.

The manuscript is organized as follows. In Section 2, we first provide relevant biological background that was necessary to construct the systems biology model. Then we describe the experimental data that was used in the study, the development of the mathematical model, and how the experimental data was integrated into numerical simulations. In Section 3, we present our main results, and finally in Section 4, we discuss the implications of our findings and potential future research directions.

## 2 Methods

We developed a multi-scale systems biology approach to model the coupling of the ISR to the intrinsic apoptosis pathway in AML. To understand how activation of the ISR helps to overcome venetoclax resistance in AML, we used the system biology approach to study the effects of combination venetoclax and tedizolid treatment on the coupled pathway, including the timing of the effects and the optimal treatment protocol. The details of the relevant biological interactions, available experimental data, the systems biology model developed here, and the numerical simulations, are explained below.

### 2.1 Relevant biology

In this section, we provide an overview of the mitochondrial (intrinsic) apoptosis pathway, how it is regulated by the c-Myc oncogene and the integrated stress response (ISR), and how these pathways affect cellular metabolism. The pathways are discussed in a context that is relevant to our study of venetoclax resistance in AML. The biological background material forms the foundation for the mathematical model and subsequent investigation. We open this section with a discussion of the intrinsic apoptosis pathway, which has received considerably more mathematical modeling attention [59–75] than the ISR pathways [76, 77].

#### 2.1.1 The Bcl-2 protein family regulates the mitochondrial apoptosis pathway

The mitochondrial (intrinsic) apoptosis pathway is regulated by the Bcl-2 family proteins through an intricate network of protein-protein interactions (PPIs) involving multiple post-transcriptional [78] and post-translational modifications [79], protein localization and trafficking processes [80], and the formation of several protein complexes. Each member of the Bcl-2 family contains one or more Bcl-2 homology (BH) domains- of which there are a total of four, and the dominant PPIs involved in the apoptosis pathway are dictated specifically by the BH3 binding domain [81, 82]. The Bcl-2 family of proteins is subdivided based upon protein structure and function into a group of (1) anti-apoptosis proteins (Bcl-2, Bcl-XL, Bcl-W, Mcl-1, Bfl-1/A1) and a group of (2) pro-apoptosis proteins, which can be further subdivided into (2-i) activators and sensitizers (Bim, Bid, Bad, Bik, Bmf, Hrk, Puma, Noxa, etc.) and (2-ii) effectors (Bax, Bak, Bok) [80, 83–86]. The anti-apoptosis and pro-apoptosis effector proteins contain all four BH domains (BH1-BH4), while the pro-apoptosis activators and sensitizers contain only the BH3 domain.

**Fig 1:**
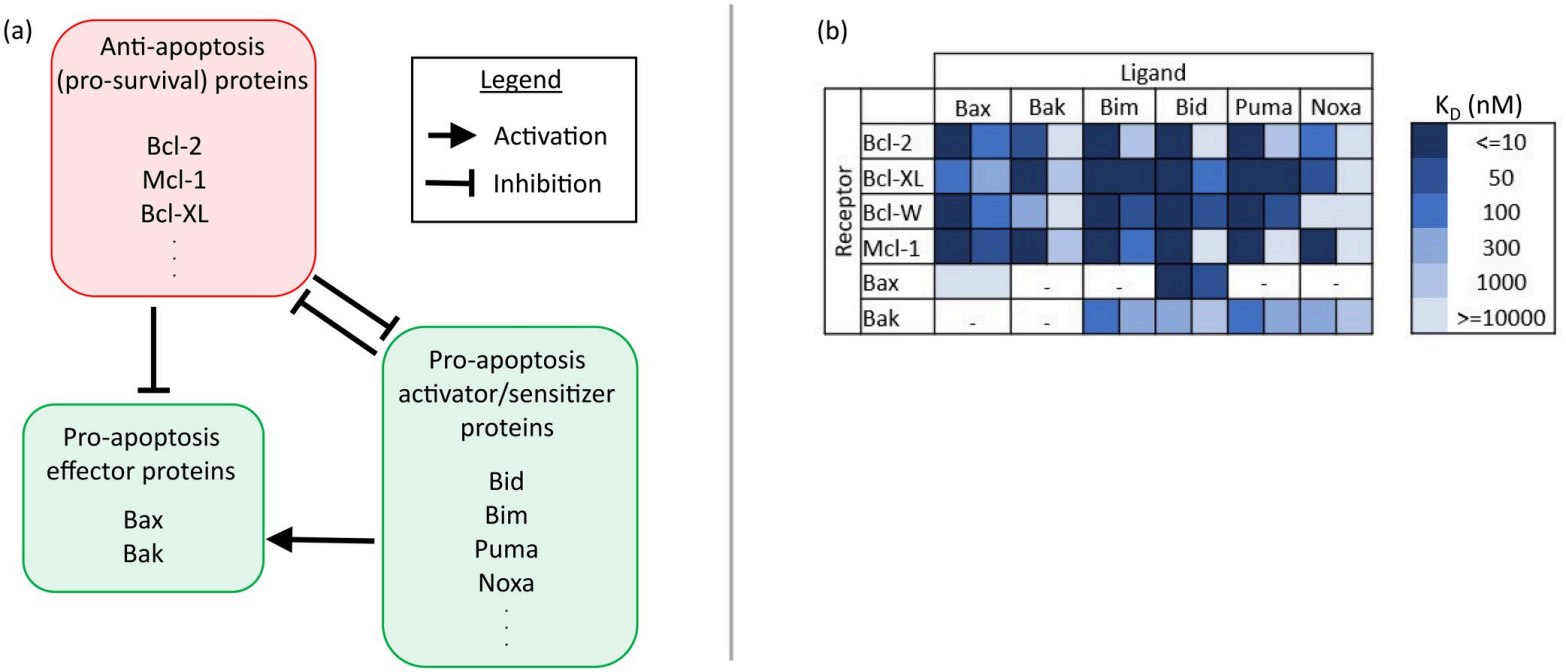
A simplified view of the intrinsic apoptosis pathway and Bcl-2 family PPI network. (a) The anti-apoptosis and pro-apoptosis proteins mutually inhibit each other as indicated. The BH3-only proteins activate the effector proteins, which go on to cause MOMP and subsequently cell death [80, 85–87, 90–99]. (b) Range of dissociation constant (*K*_*D*_) values reported in the literature for some of the BH3 peptides, truncated, and full length Bcl-2 family proteins [86]. White dashed cells indicate unreported *K*_*D*_ values for the corresponding interaction.

Competing models, reviewed in Ref. [87], describe how the Bcl-2 family protein interactions mediate mitochondrial outer membrane permeabilization (MOMP), which is considered the point-of-no-return in the mitochondrial apoptosis pathway [80]. However, it is generally accepted that the anti-apoptosis and pro-apoptosis proteins mutually inhibit each other via the formation of protein complexes with differential binding affinities [88, 89]. Specifically, the BH3-only anti-apoptosis proteins either bind to and sequester pro-survival proteins away from effector proteins, or they bind directly to effector proteins to facilitate a conformation change in the latter, activating them. The activated effector proteins form homo- and heterodimers and higher order oligomers which embed into the mitochondrial outer membrane (MOM), creating channels there. This promotes mitochondrial outer membrane permeabilization (MOMP) and the release of cytochrome c into the cytosol, which is followed by the formation of the apoptosome, the activation of several Caspases and subsequent negative feedback on the pro-survival Bcl-2 family proteins, and ultimately cell death [80, 85–87, 90–99]. A simplified view of the Bcl-2 family PPIs and their role in the intrinsic apoptosis pathway, along with experimentally reported binding affinities, is presented in Fig 1.

One of the common features of malignant cells is their ability to resist cell death [100, 101], and there are conceivably many mechanisms within the intrinsic apoptosis pathway for this to be achieved. One such mechanism is the over-expression of the Bcl-2 protein, and this is a common feature of many solid cancers and hematological malignancies and is typically associated with poor prognosis and resistance to chemotherapy. Bcl-2-specific inhibitors [5], such as venetoclax, have a high binding affinity specifically for the Bcl-2 protein. When administered, the inhibitor binds to the BH3 domain of the Bcl-2 protein, displacing a formerly bound pro-apoptosis protein. This enables the pro-apoptosis proteins to go on and fulfil their intended functions of causing MOMP and subsequent apoptosis.

#### 2.1.2 Bcl-2 inhibition impacts cellular metabolism

Research over the last few decades has indicated that the Bcl-2 protein family plays several key roles in cellular metabolism, energetics, migration, and invasion- which may be independent of its role in mitochondrial apoptosis [4, 102]. For example, experiments have shown that Bcl-2 binds to the COX-Va subunit of ETC complex-IV [103]. In leukemia cells, this leads to increased localization of the COX-Va and COX-Vb subunits to the mitochondria and subsequent enhancement of ETC complex-IV activity, which is accompanied by an increase in the overall rate of mitochondrial respiration and ROS production [4, 103–105]. In line with these findings, Bcl-2 inhibition was more recently found to exert profound effects beyond the regulation of the intrinsic apoptosis pathway. Indeed, venetoclax treatment led to the activation of several common genes in multiple mantle cell lymphoma and diffuse large B-cell lymphoma cell lines, including those involved in apoptosis, DNA damage response/repair, cell growth/survival signaling pathways, metabolism, and mitochondrial genes [102]. One such pathway is the PI3K/AKT pathway, which was activated after both short and long-term exposure to venetoclax and in acquired/intrinsic venetoclax-resistant cells, suggesting that venetoclax-exposed cells may activate this pathway as a compensatory response to promote survival and resistance [102]. In Ref. [102], venetoclax treatment led to the downregulation of key members of the glycolytic pathway in lymphoma cells. Other studies indicate that inhibiting glutamine metabolism is synergistic with Bcl-2 inhibition in leukemia and myeloma cells [106, 107], further illustrating the breadth of the link between the Bcl-2 family proteins and cancer cell metabolism [102].

In more recent work, it was found that Bcl-2 inhibition in venetoclax-resistant LCs disrupts cellular energy metabolism by causing increased mitochondrial fusion [26], which is consistent with a response to mitochondrial stress [108]. In addition, venetoclax treatment was found to have an inhibitory effect on complex-I of the electron transport chain (ETC) in resistant LCs, while having a minimal effect on ETC complexes II-IV [26]. Interestingly, a growing body of experimental evidence indicates that, in addition to their pro-apoptotic roles, Bax and Bak can anchor into the MOM in unstressed cells and bind to the ND5 subunit of complex-I, which inhibits its enzymatic activity [4, 87, 109–112]. By sequestering Bcl-2, venetoclax increases the relative abundances of free pro-apoptosis Bcl-2 family proteins and protein complexes, thus indirectly contributing to complex-I inhibition. Interestingly, by modulating the cellular metabolism, these regulatory effects lead to changes in ROS production that can subsequently influence cell migration, invasion, and metastasis [113, 114].

#### 2.1.3 The c-Myc oncogene regulates apoptosis and cellular metabolism

Over the past several decades, a tremendous amount of research has gone into understanding the role of the c-Myc proto-oncogene in directing cellular growth, proliferation, differentiation, and apoptotic signalling pathways. Given the potent role of c-Myc in regulating these processes in healthy cells, it is not surprising that c-Myc expression is tightly controlled by a complex machinery of signaling pathways, from protein synthesis to ubiquitin-mediated degradation [115, 116]. However, c-Myc expression is elevated or deregulated in a majority of human cancers, which alters global gene expression and results in tumorigenesis [117–121]. By targeting key genes involved in ribosomal and mitochondrial biogenesis, glucose and glutamine metabolism, lipid synthesis, and cell-cycle progression, the c-Myc oncoprotein contributes to the production of building blocks that enable cancer cells to grow and proliferate [117]. As cell growth increases, cellular metabolism is upregulated by the simultaneous positive effects of c-Myc overexpression on both glycolysis and oxidative phosphorylation, which include direct and indirect transcriptional changes that increase the production of glycolytic enzymes, structural and functional components of the mitochondria, and glutaminase and glutamine transporters [122–126].

As part of its regulatory effects on growth and metabolic pathways, c-Myc has been shown to upregulate eIF4E and downregulate GADD45a [127, 128]. Interestingly, GADD45a induces Bim translocation to the mitochondria [129], and the upregulation of eIF4E was observed to induce the translation of Bcl-XL [128]. Therefore, these interactions enable c-Myc to indirectly affect the intrinsic apoptosis pathway. In addition, c-Myc has also been shown to directly upregulate the transcription of Mcl-1 [128]. Furthermore, recent studies have shown that Bag-1, which markedly increases the pro-survival function of Bcl-2, is a critical, downstream target of c-Myc [130, 131]. By upregulating members of the pro-survival Bcl-2 protein family and reducing the accumulation of members of the pro-apoptotic Bcl-2 protein family at the mitochondria, these regulatory effects have a tendency to inhibit apoptotic signalling.

Paradoxically, research has shown that c-Myc expression can also trigger apoptosis. Specifically, cellular response to c-Myc appears to depend on the absolute levels or kinetic pattern of c-Myc expression, as well as the environmental conditions, including the cellular concentrations of growth factors [131]. These findings have motivated the hypothesis that cellular response to c-Myc occurs in a rheostat-like manner. Under this hypothesis, a cell transitions through states of quiescence, cell cycling, and apoptosis as c-Myc activity increases [132]. Between cell cycling and apoptosis, there exists a postulated cancer zone, wherein c-Myc promotes the initiation of cancer by upregulating cellular proliferation without accompanying differentiation [132–135]. Importantly, cancer cells acquire the ability to resist the apoptotic effects of elevated c-Myc, responding only to its proliferative signalling [131]. This is commonly accomplished through loss of surveillance mechanisms or through modifications to the expression or activity of Bcl-2 protein family members, which may be through Myc-dependent or Myc-independent mechanisms [3, 128, 131].

Beyond the cancer zone, sufficiently high expression or activity of c-Myc can trigger apoptosis. This c-Myc-dependent pattern of stimulatory and inhibitory regulation is analogous to a hormetic dose response curve. At sufficiently high concentrations of c-Myc, the balance of pro-survival and pro-apoptosis Bcl-2 protein family members is altered in favor of apoptosis, which can occur through p53-dependent or p53-independent mechanisms [131, 136]. Some of the p53-independent mechanisms that have been observed experimentally include the suppression of the expression of the pro-survival proteins Bcl-2, Bcl-XL, and Bcl-w, as well as the upregulation of the expression of the pro-apoptosis proteins Bax, Bak, Bim, Bid, and Noxa (see Refs. [136–141] and references therein).

#### 2.1.4 The integrated stress response regulates the apoptosis pathway

The integrated stress response (ISR) is an intricate, evolutionarily-conserved stress response pathway that is present in eukaryotic cells, whose primary role is to restore cellular homeostasis. The EIF2*α* kinases are the first responders to cellular stress signals, and the precise physiological and/or pathological conditions determine which of the kinases are activated [142–147]. While global protein synthesis is attenuated early in the stress response, proteins that contain short upstream open reading frames (uORFs) in their 5’ untranslated region (5’UTR), such as the transcription factor ATF4, are preferentially translated to aid in cell survival and recovery [145]. The transcription factor ATF4 induces the transcription of several stress response genes, including GADD34 and Chop, which both contain a 5’ uORF [148, 149].

While GADD34 restores global protein synthesis, in a delayed response to prolonged conditions of cellular stress, ATF4 also transcriptionally activates 4E-BP. This pathway is a recently proposed second node of global translational inhibition in the ISR that shifts the cellular response toward cap-independent translation [148]. This results in increased translation of Chop and other stress response and apoptosis proteins [150, 151], as well as proteins required for regulatory cell processes, such as c-Myc [148]. During sustained or heightened activation of the ISR, the transcription factor Chop induces apoptosis by upregulating the expression of pro-apoptosis proteins and repressing the expression of pro-survival proteins [145, 146, 149]. Specifically, Chop has been reported to repress the expression of Bcl-2, Bcl-XL, and Mcl-1, and upregulate the expression of Bim, Bid, Bik, Puma, Bax, Bak, and Bad through both direct and indirect transcriptional and post-transcriptional events [143, 152–157].

Previous work [26] found that both venetoclax and tedizolid individually lead to a transient increase in Chop expression, reflecting a sub-lethal activation of the ISR, in venetoclax-resistant MOLM-13 AML cells. Moreover, their combination leads to a heightened stress response, characterized by a further increase in Chop expression that is sufficient to trigger cellular apoptosis. In the case of venetoclax monotherapy in venetoclax-resistant MOLM-13 cells, treatment-induced mitochondrial dysfunction, see Section 2.1.2, is likely to promote activation of the ISR. On the other hand, tedizolid is an oxazolidinone-class antibiotic that works by inhibiting mitochondrial protein translation [26, 158]. In venetoclax-resistant MOLM-13 cells, tedizolid monotherapy led to several mitochondrial alterations, including inhibition of mitochondrial respiration and changes in the mitochondrial ultrastructure that are consistent with mitochondrial stress [26]. In combination with venetoclax treatment, the tedizolid-induced disruption of mitochondrial translation could halt the adaptive transcriptional changes that occur with venetoclax monotherapy, leading to a heightened stress response and potentially the activation of additional stress response pathways. Importantly, due to the cross-talk between the mitochondria and endoplasmic reticulum (particularly via calcium signalling), stress in one of these membrane-bound organelles can trigger stress or dysfunction in the other [159–161], potentially activating other specific stress response pathways in parallel, causing a heightened ISR.

### 2.2 Experimental data

The experimental data set used in this work is comprised of in vitro measurements of the oncogene c-Myc, the ISR effector protein Chop, the Bcl-2 family proteins Bcl-2, Mcl-1, Bim, Bax, Bak, and the executioner of apoptosis active Caspase-3 levels, in a line of venetoclax resistant AML cells, MOLM-13 R2, under different treatment conditions [26]. The c-Myc protein levels in treated cells were measured using intracellular flow cytometry analysis as previously described using an Alexa Fluor 647 conjugated anti-MYC antibody (Cell Signaling Technology, Cat # 13871). Chop protein expression was estimated by Chop reporter expression [26] and Chop mRNA levels. Chop mRNA levels in treated cells were measured using quantitative RT-PCR as previously described [26] using validated primers (OriGene, Cat # HP207450). Bcl-2, Mcl-1, Bim, Bax, Bak, and active Caspase-3 protein expression levels were measured by Western blotting, as previously described [26].

Due to experimental limitations, the resulting data set does not provide quantitative measurements of the initial concentration of the proteins c-Myc, Chop, Bcl-2, Mcl-1, Bim, Bax, Bak, or active Caspase-3 in untreated cells. Rather, the expression of c-Myc, relative to its initial concentration, was measured at *t* = 24, 48, and 72 hours post treatment administration, while the relative expression of Chop was measured at *t* = 24, 48, 72, and 96 hours post treatment administration. The relative expressions of Bcl-2, Mcl-1, Bim, Bax, Bak, and active Caspase-3 were measured at *t* = 72 hours post treatment administration.

In addition to protein expression levels, cellular proliferation and cell viability was also measured under each treatment condition for the MOLM-13 R2 cells at *t* = 24, 48, 72, 96, and 120 hours post treatment administration. The number of viable cells at each time point was determined by Annexin V staining and counting beads, as previously described [26].

The treatment conditions consisted of: single dose venetoclax monotherapy (400 nM concentration), single dose tedizolid monotherapy (5 *μ*M concentration), and single dose venetoclax and tedizolid combination therapy (400 nM and 5 *μ*M concentration, respectively), with the drugs administered simultaneously. No drug washout was performed during experiments, and the drugs were not replenished over the five day window. The experiments were repeated three times, and the average and standard deviation of all expression levels, cell viability measurements, and live cell numbers were reported.

To study the optimal drug timing predicted by the systems biology model, venetoclax resistant MOLM-13 cells were pre-treated with tedizolid at 5 *μ*M or DMSO for 2 days. After the pre-treatment phase, the cells were washed and treated with tedizolid (5*μ*M) in combination with venetoclax at different concentrations from 10 nM to 10 *μ*M. The number of viable cells after 3 days of combination treatment was determined by Annexin V and counting beads.

### 2.3 Integrative mathematical model

Guided by the available experimental data (RNA and protein expression, cell viability, and cellular proliferation), see Section 2.2, along with the previously reported relevant biological interactions, see Section 2.1, we developed a systems biology approach to model cellular drug uptake and the resulting on-target and off-target drug effects of venetoclax and tedizolid that are linked to the intrinsic apoptosis pathway and the ISR pathway. The model consists of a system of 28 coupled ordinary differential equations (ODEs) that capture drug uptake, the dominant effects of the drugs on protein expression, the regulatory effects of transcription factors on the Bcl-2 family protein levels, the interactions between the Bcl-2 family proteins, and the activation of Caspase-3 due to the Caspase cascade, which ultimately leads to cellular apoptosis.

Both cellular proliferation and cell death were modeled in this work, guided by the reported interactions in the literature. The timescales associated with drug uptake, protein level changes, and protein binding (hours) are different from the timescale of changes in the cellular populations due to cellular division, differentiation, and death (days). This separation in timescales gives rise to a multi-scale mathematical model, which we calibrate with the multi-modal experimental data described in Section 2.2. The details of the mathematical model, including all of the equations and kinetic parameter values, are presented in S1 Text. Here we give an overview of the different cellular processes and molecular interactions in the model, then discuss how numerical simulations and sensitivity analysis were performed.

**Fig 2:**
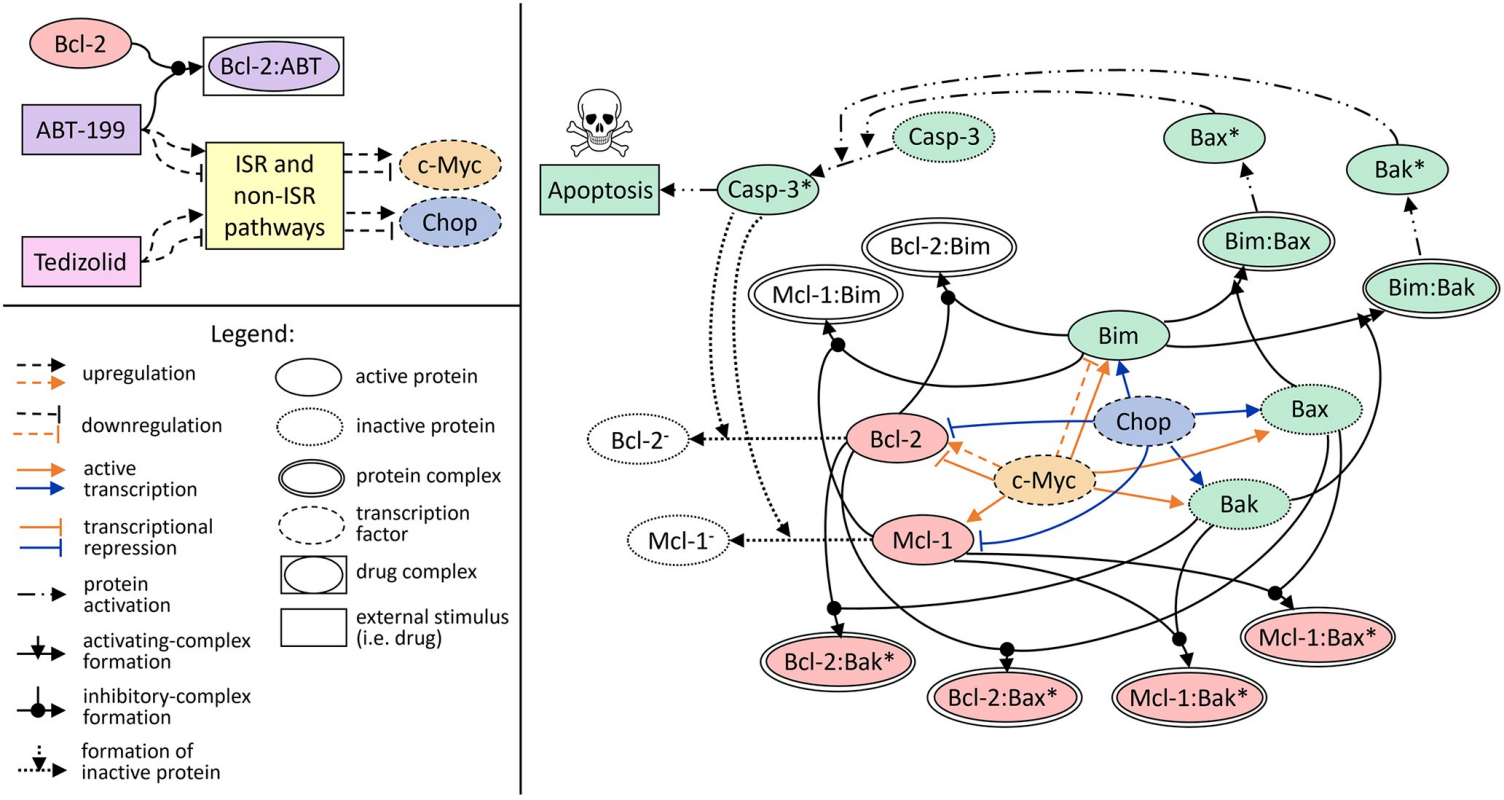
Protein network corresponding to the key interactions between the intrinsic apoptosis pathway and the integrated stress response (ISR). Direct and indirect regulatory effects of venetoclax (ABT-199) and tedizolid on the coupled pathway are shown in the left panel. ABT-199 binds to Bcl-2 to form a protein-drug complex [5, 162]. In addition, both ABT-199 and tedizolid perturb ISR-related and non-ISR pathways, ultimately exerting both positive and negative regulatory effects on c-Myc and Chop (ISR effector protein) expression levels [26, 116]. The coupled apoptosis-ISR pathway is shown in the right panel and depicts the regulatory effects of the transcription factors c-Myc and Chop on the Bcl-2 family proteins, including both the pro-survival and pro-apoptosis effects of c-Myc [128, 130–132, 136–141], as well as the pro-apoptosis effects of Chop [143, 145, 146, 149, 152–157]. In addition, the interactions between the Bcl-2 family proteins [87–89] are shown, including the binding of anti-apoptosis proteins (Bcl-2, Mcl-1) with pro-apoptosis activator (Bim) and effector (Bax, Bak) proteins, as well as the binding of activator and effector proteins and the subsequent activation of the latter. The activated effector proteins cause Caspase-3 activation, which causes cleavage of the anti-apoptosis proteins and promotes apoptosis [80, 85–87, 90–99]. The pro-apoptosis species are coloured green, anti-apoptosis species are red, neutral species are white, c-Myc is orange, and Chop is blue.

We present the biological pathway that underlies the mathematical model used in this work in Fig 2. The biochemical reaction network was converted to a set of 28 coupled ODEs using the law of mass action. Briefly, we incorporate cellular drug uptake and drug decay as previously described [116], see Section A.1 in S1 Text for a brief summary. The model incorporates the inhibitory effects of venetoclax on the Bcl-2 protein (see Section 2.1.2), including its sequestration from the pro-apoptosis proteins and subsequent binding to form the protein-drug complex [162]. This is accomplished by incorporating a higher binding affinity between Bcl-2 and venetoclax than between Bcl-2 and the pro-apoptosis proteins, see Section A.5 in S1 Text.

The systems biology approach also incorporates the effects of venetoclax and tedizolid on the regulatory networks of the transcription factors c-Myc and Chop, which arise from the modulation of both ISR and non-ISR related pathways [26, 116]. To model these regulatory interactions, we implemented our previously developed mean-field model [116] for c-Myc and Chop, which captures the dominant drug-induced regulatory effects on the expression of both proteins and allows for the inclusion of temporal delays for downstream effects. The mean-field model was calibrated using the expression levels of c-Myc and Chop at *t* = 24, 48, and 72 hours post treatment administration under each treatment condition described in Section 2.2. As described in Ref. [116], the predictions of the model were validated with RNA sequencing data that was collected at *t* = 96 hours after treatment administration under each treatment condition. The RNA sequencing data provided a snapshot of the gene expression profiles for all the species comprising the regulatory networks of c-Myc and Chop, giving context to the dominant drug effects predicted by the mean-field model. We direct the reader to Section A.1 in S1 Text and Ref. [116] for further details regarding the ODEs used to model cellular drug uptake and the expression of c-Myc and Chop, including the kinetic parameter values and temporal delays.

In addition to the on-target drug effects, by modulating the expression of c-Myc and Chop, both venetoclax and tedizolid treatment result in broad cellular-level changes. For instance, as outlined in Sections 2.1.3 and 2.1.4, the transcription factors c-Myc and Chop both exert significant regulation over the intrinsic apoptosis pathway by directly controlling the expression of the Bcl-2 family proteins. In Fig 2, we depict the previously reported regulatory interactions that are incorporated into the model. These regulatory interactions, along with their corresponding rate laws, are further described in Section A.2 in S1 Text. We note that this is the predominant mechanism by which the ISR pathway is coupled to the intrinsic apoptosis pathway in this work.

The model also includes the interactions between the Bcl-2 family proteins, see Section 2.1.1, including the formation of protein complexes between the anti-apoptosis proteins and pro-apoptosis proteins, as well as pro-apoptosis activator and pro-apoptosis effector protein complex formation and the subsequent activation of the pro-apoptosis effector proteins. Each individual interaction, along with the corresponding rate law, is described further in Section A.3 in S1 Text. In addition, the model includes the activation of Caspase-3 and the subsequent feedback of active Caspase-3 on the anti-apoptosis proteins, see Section 2.1.1. These individual interactions and their corresponding rate laws are described further in Section A.4 in S1 Text.

To reduce the number of kinetic parameters due to limited data availability, as indicated by Fig 2, only a subset of the Bcl-2 family proteins is included in the mathematical model. Particularly, only the most highly expressed Bcl-2 family proteins for which treated expression levels were measured, see Ref. [26], were modeled. By this criteria, we justified the exclusion of Bcl-2 family proteins with relatively lower expression in the resistant cells, especially if their signaling in the intrinsic apoptosis pathway has redundancies, such as Bcl-XL and Bid. Referring to Fig 1, we see that Bcl-XL and Mcl-1, for example, have redundant functions in the intrinsic apoptosis pathway, and both proteins are not sequestered by venetoclax, since venetoclax binds specifically to Bcl-2. Moreover, Bcl-XL expression was lower in the MOLM-13 R2 cell line than in the parental cell line, while Mcl-1 expression was significantly (over 40 times) higher in the resistant cell line compared to the parental cell line, indicating that Mcl-1 played a much more significant role in the development of drug resistance to venetoclax in these cells. Due to such redundancies and differences in protein expression, we expect the inclusion of only the dominant protein-protein interactions in the model not to significantly impact the results.

The metabolic effects of the drugs, see Sections 2.1.2 and 2.1.4, and their subsequent impact on cellular proliferation, were also incorporated into the model. For venetoclax, the metabolic changes were modeled by including a term in the cellular proliferation that depends on the concentration of free Bcl-2 protein. This way, when venetoclax is administered and sequesters the Bcl-2 protein, the concentration of free Bcl-2 decreases, which disrupts cellular metabolism in the model, consistent with what has been observed experimentally [26]. For tedizolid, since the metabolic effects were not seen to depend directly on any specific protein in the pathway in Fig 2, we included an inhibitory term on cellular proliferation that depends on the drug concentration itself. Furthermore, the model includes a term in the cellular proliferation that depends on c-Myc concentration to account for the regulatory role of c-Myc over-expression in glycolysis and oxidative phosphorylation, see Section 2.1.3. Finally, we modelled the rate of cell death to depend on the fraction of active Caspase-3 in the system. The net proliferation of cells was then taken to depend on the difference between the rate of cellular proliferation and the rate of cell death, as described further in Section A.6 in S1 Text.

### 2.4 Numerical simulations

We integrated the available multi-modal experimental data into our multi-scale mathematical model to study the role of the integrated stress response in overcoming venetoclax resistance, as well as the timing of the cellular decision to commit to apoptosis. Of the 87 kinetic parameters related to Bcl-2 regulation, 34 were identified in the literature and fixed at previously measured values in numerical simulations, see Tables E and F in S1 Text. The remaining 53 kinetic parameter values were estimated by simulating the in vitro treatment protocols described in Section 2.2 and forcing the simulated protein levels, cellular proliferation, and cell viability to match to the experimental data using the MATLAB genetic algorithm. To prevent over-fitting to the 72 data points (24 cell viability, 24 cell counts, 24 protein expression), the simulated results were taken to be equivalent to the experimental data if they agreed within (typically) one standard deviation of the mean. Outside of this range, the loss function for a particular data point was calculated as the absolute relative error:

$$\begin{array}{c} f\left(x_{s}\right)=\{\begin{array}{cc} \left(x_{s}-x_{+}\right)/x_{+}, & \text{if}\;x_{s}>x_{+}=\bar{x}+\Delta x,\\ \left(x_{-}-x_{s}\right)/x_{-}, & \text{if}\;x_{s}<x_{-}=\bar{x}-\Delta x. \end{array} \end{array}$$ (1)

In Eq (1), *x*_*s*_ is a simulated data point and $\bar{x}$ is the mean value of the corresponding experimental measurement and Δ*x* the associated standard deviation. For a subset of the experimental data, such as cell number or cell viability measurements with small coefficient of variation, it was helpful to begin fitting within two or more standard deviations of the mean. Then, the lower and upper bounds, *x*_−_ and *x*_+_, were slowly decreased to a single standard deviation during the fitting process. After calculating *f*(*x*_*s*_) for all *x*_*s*_, the absolute relative errors for all the protein measurements were summed to calculate *f*_*p*_, and similarly, *f*_*v*_ was calculated as the sum of absolute relative errors for the cell viability data and *f*_*c*_ for the cell numbers. Finally, the objective function was taken to be a weighted sum of *f*_*p*_, *f*_*v*_, and *f*_*c*_. To ensure that the weighted contributions of *f*_*p*_, *f*_*v*_, and *f*_*c*_ to the objective function were approximately the same order of magnitude (enabling the model to simultaneously capture the trends in all data sets), the relative contributions were approximately taken to be inversely proportional to the coefficients of variation of the data. In particular, the relative contribution of *f*_*p*_, *f*_*v*_, and *f*_*c*_ to the objective function was typically taken to be 1 : 0.1 : 0.1 or 1 : 0.2 : 0.05, respectively. Toggling between two sets of weightings enabled the model to initially get a better match to a subset of the data, and then increase the importance of the fit to the remaining data in a semi-modular fashion.

During the model calibration process described above, the search space for the kinetic parameters and initial conditions was set to previously reported biologically-relevant ranges where available (see Section B in S1 Text for details). For parameters such as, e.g., Hill coefficients, half-saturation constants, protein production rates, and previously unreported protein half-lives, narrow search ranges (i.e. soft boundaries) were implemented at the start of the calibration process. After a block of ∼25–100 successive iterations of the genetic algorithm, the search ranges were altered (widened, shifted, etc.) if a parameter value tended toward a particular endpoint of the soft boundary. During this process, biological relevancy was maintained by ensuring that the updated search ranges (i.e. soft boundaries) did not conflict with known biologically-relevant hard boundaries (e.g. most protein half-lives should be on the order of minutes to roughly 24 hours, Hill coefficients for sharp response functions are given strict upper cut-offs to minimize numerical error, protein production rates are limited by steady state assumptions, see Table G in S1 Text, etc.). Early in the calibration process, the soft boundaries were adjusted by larger amounts, such as up to 10% (if it was still within the hard boundary). Later in the calibration process, if the objective function did not change significantly over blocks, parameter search ranges were narrowed or widened in a similar manner. In the late stages of calibration, as the model converged on the optimal parameter set, the soft boundaries were adjusted by smaller amounts, such as, e.g. 1% or less.

During calibration, the “CrossoverFraction” was set to 0.5 for the first ∼15–25 iterations of each block, then it was set to 0.9 for the remaining iterations. This enabled the algorithm to search partially at random through mutations early on, while searching primarily via crossover for the remaining iterations. The MATLAB dde15s solver [163] was used to integrate the system of coupled ODEs (see Data availability statement). This solver modifies the MATLAB dde23 solver to determine solutions to stiff delay differential equations by the method of steps with ode15s as the integrator. Due to the different timescales in the mathematical model, to increase the efficiency of numerical simulations, the system of coupled ODEs was non-dimensionalized in our code and units were restored when interpreting the results.

The search for a kinetic parameter set was accomplished in two steps. First, the kinetic parameters related to drug uptake and clearance, as well as the regulatory effects of the drugs on the transcription factors c-Myc and Chop, were determined. This was accomplished by developing a mean-field approach [116] to simulate the effects of the three treatment protocols described in Section 2.2 on the expression of c-Myc and Chop. For consistency with experiments, the drugs were administered either individually or in combination, at the beginning of the simulations, and no drug washout or replenishment was administered over the course of the simulation. By forcing the expression of the transcription factors to match to the experimental measurements on days 1, 2, and 3 for c-Myc and on days 1, 2, 3, and 4 for Chop, under all treatment conditions, a set of kinetic parameters was determined for step one, see Table D in S1 Text. Using this kinetic parameter set, the simulated protein expression levels matched to the average experimental data with a relative error of 0.03, calculated over all time points and treatment conditions. We then investigated the RNA sequencing data that was collected on day 4 of experiments and found that the predictions of the mean-field model were justified by the RNA-level trends, see Ref. [116]. Particularly, the drug uptake predicted by the mean-field model was consistent with cellular-level changes suggested by the RNA-sequencing data in the resistant versus parental cell line. In addition, the dominant regulatory effects of the drugs on the expression of c-Myc and Chop were justified by the RNA-level changes to their individual regulatory networks.

After obtaining a match to the transcription factor data, we next simulated the entire pathway, including the regulatory effects of the transcription factors on the Bcl-2 family protein levels. During this process, we set the 31 drug and transcription factor parameters to the values that were determined in the first step. We then searched for the remaining parameter values by simulating the different treatment protocols and forcing the simulated total expression of Bcl-2, Mcl-1, Bim, Bax, Bak, and active Caspase-3 on day 3 to match to the experimental data for all treatment conditions. In addition, we forced the simulated cell viability and cellular proliferation on days 1, 2, 3, 4, and 5 to match to the experimental data for all treatment conditions and for the untreated case, as explained above.

Given that the protein interactions happen on a faster time scale than cellular division, we followed the work of Ref. [71] and assumed the quasi-steady state for protein expression when initializing the simulations. Specifically, we first turned off all Bcl-2 family protein-binding interactions and calculated the maximal production rates for each Bcl-2 family protein such that the initial abundance of the free Bcl-2 family protein levels balanced the production and degradation. We then ran an equilibrium simulation, where the free Bcl-2 family protein levels were set to the initial abundance, and all protein complexes, inactive anti-apoptosis protein levels, active pro-apoptosis levels, and active Caspase-3 levels were initialized to zero. We turned the Bcl-2 family protein-binding interactions back on, and allowed the system to evolve until it settled to a steady state. In this steady state, the formation and dissociation of protein complexes was balanced, so that the concentration of all species in the system was constant in time. This was considered to be the state of the untreated system [71, 72]. We used this untreated steady state as the initial state for all treatment simulations.

The kinetic parameter set obtained this way and used throughout the work is presented in Table G in S1 Text. We note that, due to the complexity of the model and the limitations in data availability, there could be several sets of kinetic parameters that fit equally well to the experimental data. However, we previously found that the 31 kinetic parameters in the first step of the model calibration fit within only a particularly narrow range of values [116]. For this reason, we would not expect to obtain a different set of values for these 31 kinetic parameters if the fitting approach had been combined into a single step. Furthermore, since we found the time complexity of the fitting approach to scale nonlinearly with the dimensionality of the search space, it was more computationally efficient to calibrate the model with a multi-step approach.

### 2.5 Sensitivity analysis

Local sensitivity analysis was conducted around the nominal parameter set to determine how small perturbations to the parameter values affect the strength of the response to treatment. This enabled the determination of which interactions in the model have a dominant effect on the system dynamics. To this end, we perturbed the kinetic parameter values and initial conditions one-at-a-time by ±1% and calculated the resulting sensitivity as follows:

$$\begin{array}{c} S=\frac{\Delta v/v_{0}}{\Delta p/p_{0}}. \end{array}$$ (2)

In this equation, *v*_0_ is the cell viability on day five corresponding the nominal (unperturbed) parameter value (or initial condition) *p*_0_ and Δ*v* is the change in cell viability on day five resulting from the perturbation Δ*p* to the parameter value (or initial condition). In this way, a relative change in the cell viability that is less than the relative change in parameter value (or initial condition) corresponds to a local sensitivity of less than one. On the other hand, if the relative change in the cell viability is greater than the relative change in the parameter value (or initial condition), then the local sensitivity will be greater than one. The latter case indicates that the system dynamics is sensitive to the corresponding interaction, which a higher local sensitivity value correlating to a more dominant role. For completeness, a global sensitivity analysis was also conducted for the model parameters, as described further in Section C in S1 Text.

### 2.6 Optimal drug timing

Using the nominal kinetic parameter set, we performed several numerical simulations to investigate the optimal timing and order of drug administration. We examined the effects of pre-treatment with venetoclax prior to the administration of combination therapy as well as tedizolid pre-treatment prior to administration of combination therapy. We also examined how the size of the pre-treatment window (i.e. the delay between the start of pre-treatment and the administration of the combination therapy) in both cases changes the response to combination treatment. To this end, we defined a quantity called the response, which is given by:

$$\begin{array}{c} R\left(\tau \right)=(\frac{N_{0}\left(t_{n}\right)}{N_{\tau }\left(t_{n}\right)})(\frac{{\text{max}}_{i=1\ldots n}N_{0}\left(t_{i}\right)}{{\text{max}}_{i=1\ldots n}N_{\tau }\left(t_{i}\right)}), \end{array}$$ (3)

where *N*_0_(*t*_*i*_) is the live cell count at time point *t*_*i*_ during combination treatment without pre-treatment, *N*_*τ*_(*t*_*i*_) is the corresponding quantity with a pre-treatment window of *τ* ∈ [1, 96] hours, *n* is the number of time points in the simulation (with *n* = 25, 000 outputs over a 5-day simulation window and increased proportionally to the pre-treatment delay), and the first time point *t*_*i*=1_ corresponds to the time at which the combination therapy is administered.

The first term in the response compares the endpoint cell numbers and is >1 if the endpoint cell numbers with pre-treatment are smaller than the endpoint cell numbers without pre-treatment. The second term in Eq (3) is the ratio of the maximal values of live cells during the combination treatments. This term will be >1 if the pre-treatment leads to a smaller peak value in live cells compared to the combination therapy without pre-treatment. Including both of these terms into the response ensures that the pre-treatment combination therapy is considered more effective if it leads to less cancer cells at the end of the treatment window *and* if it does not lead to a significantly larger spike in the number of cancer cells during the treatment. Since the dead cells are directly coupled to the live cell population, see Eq. (A.16) in S1 Text, this ensures there will not be massive cancer cell death over a short window, which, in clinical practice is a common and severe complication of induction therapy in AML patients known as tumor lysis syndrome [164].

## 3 Results

### 3.1 Mathematical model captures trends in protein expression, cellular viability, and cellular proliferation

**Fig 3:**
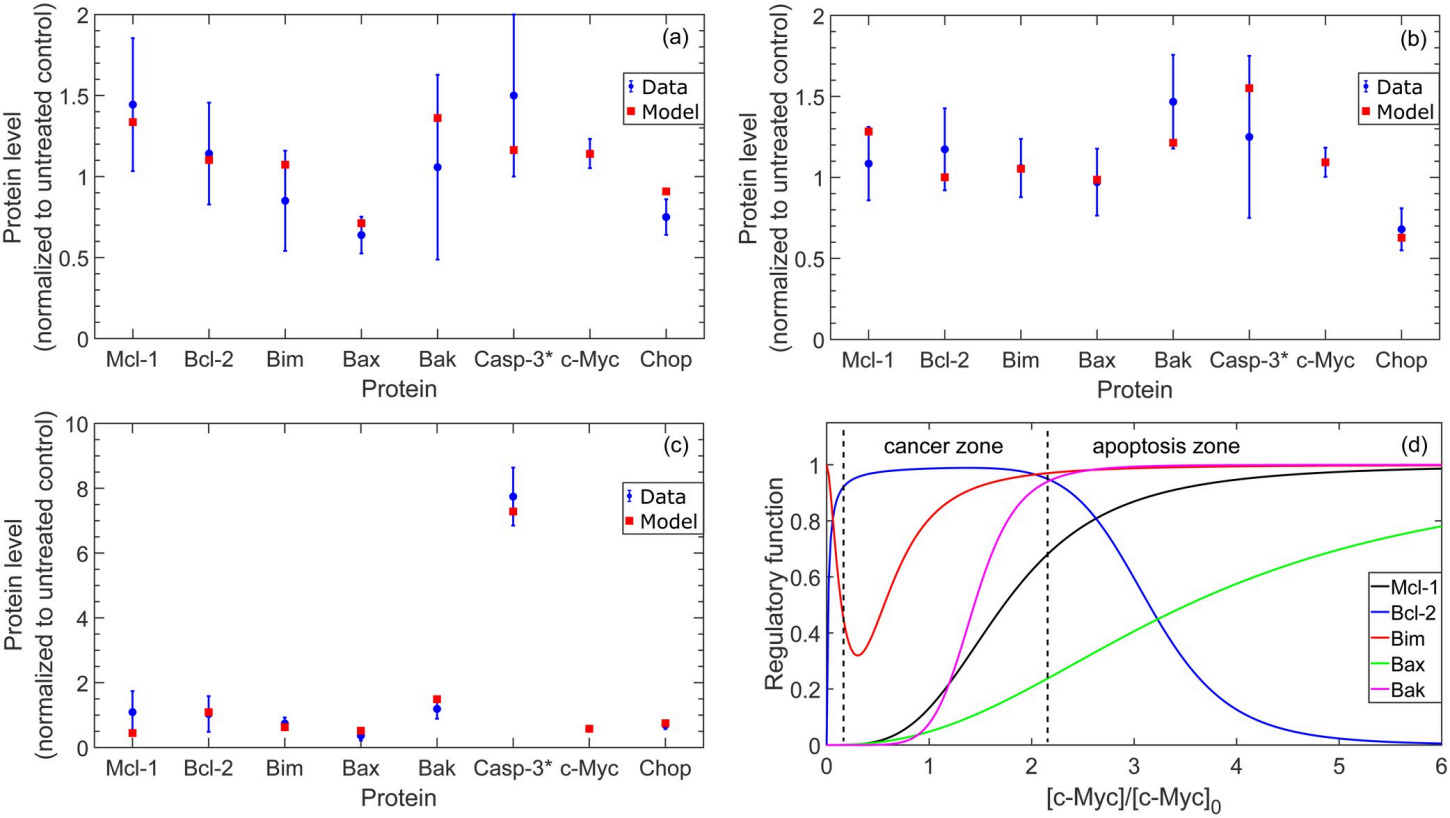
Simulated protein expression levels at *t* = 72 hour in comparison to experimentally-measured protein expression in MOLM-13 R2 cells. Blue circles correspond to the average of experimental measurements and error bars correspond to one standard deviation. Plots illustrate total protein expression levels for Mcl-1, Bcl-2, Bim, Bax, Bak, c-Myc, and Chop, and active Caspase-3 levels only. All protein levels are normalized to the expression in the untreated system. (a) 400 nM venetoclax monotherapy, (b) 5,000 nM tedizolid monotherapy, (c) 400 nM venetoclax and 5,000 nM tedizolid combination therapy administered simultaneously. (d) Shows the predicted c-Myc-dependent regulatory functions for each Bcl-2 family protein in the model, normalized by the transcription factor-dependent maximal production rate for each protein. The boundaries separating distinct zones are illustrative and were chosen as approximately the concentration of c-Myc that separates states where pro-apoptotic c-Myc-dependent regulatory functions are predominantly saturated from states where the anti-apoptotic c-Myc-dependent regulatory functions are predominantly saturated (see Eqs. (A.18)-(A.22) in S1 Text).

In Fig 3, we present the simulated protein levels at *t* = 72 hours in comparison with the experimentally measured protein expression. In this figure, the simulated protein levels were obtained from the mathematical model with the nominal kinetic parameter set presented in S1 Text. As illustrated in the figure, the mathematical model captures the changes in the total expression of the Bcl-2 family proteins under all treatment conditions. Importantly, the model reproduces the modest changes in active Caspase-3 levels with venetoclax and tedizolid monotherapies, as well as the significant increase in active Caspase-3 levels observed with combination treatment.

In Fig 3D, we also present the predicted c-Myc-dependent regulatory functions for each Bcl-2 family protein in the mathematical model, corresponding to the nominal kinetic parameter set. We note that the regulatory functions in the figure have been normalized by the transcription factor-dependent maximal production rates for each protein, see Eq. (B.1) and Table G in S1 Text. This way, the regulatory functions are scaled to lie within the same range, i.e. [0, 1], enabling a comparison of the shapes of the regulatory functions at different concentrations of c-Myc. Importantly, we see from the figure that the regulatory functions predicted by the mathematical model are consistent with the hormetic-like dose response that has been previously hypothesized for c-Myc, see Section 2.1.3. Specifically, the model predicts that there is a range of c-Myc concentrations, the hypothesized “cancer zone”, wherein the predominant function of c-Myc is to promote cell growth, as well as a range of c-Myc concentrations, the hypothesized “apoptosis zone”, wherein the primary function of c-Myc is to promote cell death.

**Fig 4:**
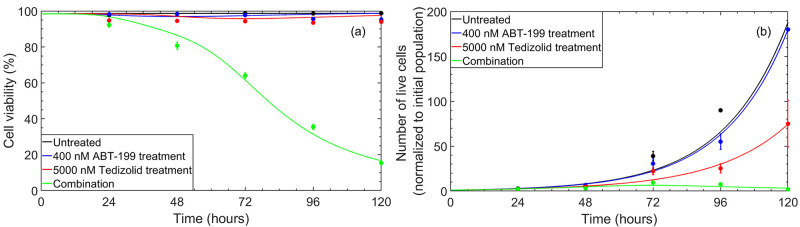
Cellular viability (a) and live cell population (b) in untreated and treated MOLM-13 R2 cells. Solid lines show the results of the mathematical model, while data points correspond to the average of experimental measurements over the five-day window. Error bars correspond to one standard deviation obtained from experimental measurements.

We present the cellular viability and cellular proliferation results in Fig 4, which illustrate an excellent qualitative agreement between the mathematical model and the experimental measurements. Particularly, the mathematical model captures the relatively constant cellular viability trends that are observed in the untreated and monotherapy cases, as well as the smooth decline in cellular viability over the five-day window with 400 nM venetoclax and 5,000 nM tedizolid combination treatment. It should be noted that the model does not fully capture the *quantitative* trends in cellular proliferation, particularly for the untreated case. In this case, the average number of live cells observed on day five of experiments was identical to the average number of live cells observed on day five for the case of 400 nM venetoclax monotherapy. Examination of the experimental data points reveals that the cellular proliferation deviates slightly from exponential growth in the untreated case and in the case of 5,000 nM tedizolid monotherapy. Since the mathematical model assumes an exponential growth rate that depends on the protein expression levels, these deviations cannot be quantitatively captured by the model. Nevertheless, the mathematical model provides excellent qualitative agreement, reproducing the overall trends for each treatment condition.

### 3.2 Effectiveness of combination therapy relies on the activation of the ISR

**Fig 5:**
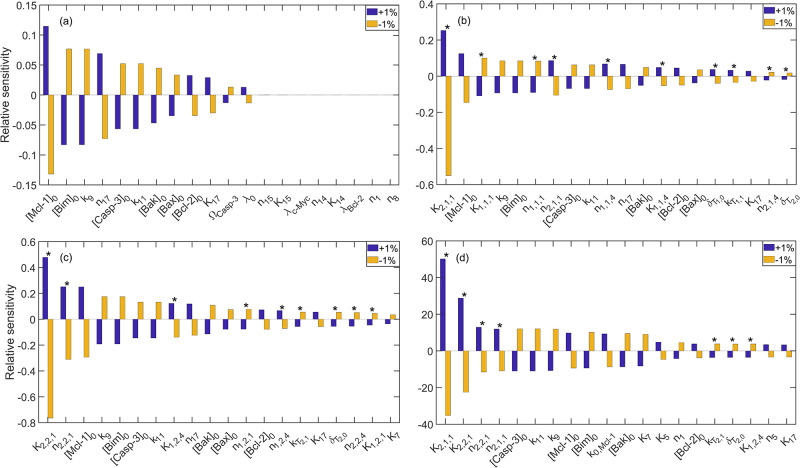
Results of local sensitivity analysis around the nominal parameter set for (a) the untreated system, (b) 400 nM venetoclax monotherapy, (c) 5,000 nM tedizolid monotherapy, and (d) 400 nM venetoclax and 5,000 nM tedizolid combination therapy. Plots show the relative sensitivity calculated using Eq (2) resulting from perturbations of ±1% to individual kinetic parameters or initial conditions in each treatment case. Top 20 kinetic parameters/initial conditions are shown in each plot. Asterisks denote kinetic parameters that affect the expression of the transcription factors c-Myc and Chop (see Ref. [116]).

We performed local sensitivity analysis around the nominal parameter set, as described in Section 2.5, to determine the dominant mechanisms underlying the response to each treatment condition in the MOLM-13 R2 cells. In particular, we perturbed each kinetic parameter and initial condition presented in Table G in S1 Text one-at-a-time by ±1% and calculated the corresponding relative sensitivity using Eq (2). The results are shown for the untreated system, 400 nM venetoclax monotherapy, 5,000 nM tedizolid monotherapy, and 400 nM venetoclax and 5,000 nM tedizolid combination treatment in Fig 5 and tabulated in Table H in S1 Text. As indicated in Fig 5, for the untreated system and for both monotherapies, the system response is highly robust to perturbations in the parameter values and initial conditions. Specifically, in the untreated system, perturbations of 1% lead to (at maximum) less than 0.15% change in cellular viability. In the cases of venetoclax and tedizolid monotherapies, perturbations of 1% lead to (at maximum) less than 0.6% and 0.8% changes in cellular viability, respectively. Furthermore, as Fig 5 illustrates, the initial concentration of Mcl-1 is the most sensitive quantity in the untreated system, while the half-saturation constants corresponding to the drug-induced production of Chop are the most sensitive parameters for both monotherapies.

In contrast to the results for the untreated system and for the individual therapies, the response to combination treatment is highly sensitive to perturbations in a subset of the kinetic parameter values. In particular, a perturbation of 1% leads to a greater than 40% change in the cellular viability for the top kinetic parameter in the case of combination therapy. Importantly, as illustrated in Fig 5, the top four kinetic parameters underlying the response to combination treatment are related to the drug-induced expression of Chop protein. Furthermore, six of the top 20 most sensitive quantities are related to the expression of Chop, which indicates that the ISR plays a crucial role in the response to combination treatment. Interestingly, while the parameters that control the expression of Chop protein were found to be most significant, the response was also found to be sensitive to kinetic parameters that control the regulation of Bak, Bim, and Mcl-1 by c-Myc. We note that these results are supported by global sensitivity analysis (see Section C in S1 Text).

The results of local sensitivity analysis indicate that the activation of the ISR is indeed highly important for the favourable outcome of combination therapy, and in particular, for overcoming resistance to venetoclax treatment. However, the precise mechanism(s) underlying the response to combination treatment is still not clear from these results. Since Mcl-1 upregulation is the dominant mechanism of drug resistance to venetoclax in the MOLM-13 R2 cell line, we next sought to understand whether Mcl-1 inhibition is the predominant mechanism by which ISR activation helps to overcome venetoclax resistance. To this end, we simulated a combination therapy consisting of 400 nM venetoclax treatment administered simultaneously with an Mcl-1 inhibitor. The Mcl-1 inhibitor was simulated to follow the same kinetic pattern as the inhibitory regulation of Mcl-1 by Chop in the combination venetoclax/tedizolid treatment.

**Fig 6:**
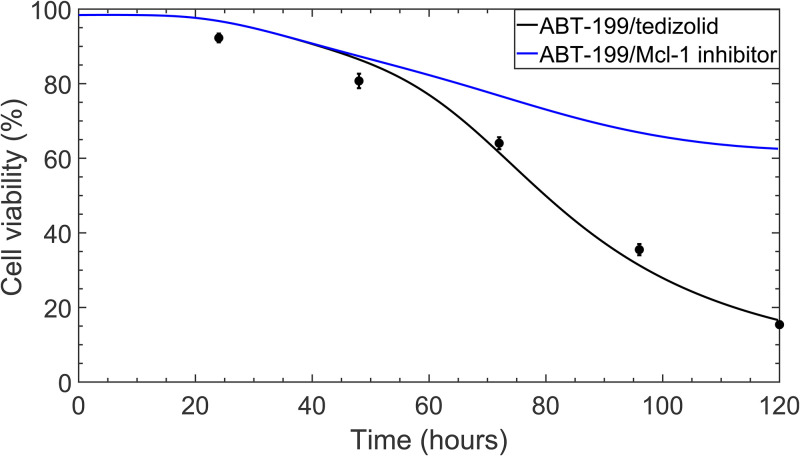
Cellular viability for 400 nM venetoclax treatment in combination with either 5,000 nM tedizolid treatment or an Mcl-1 inhibitor. Solid lines indicate the results of the mathematical model. Experimental data points for venetoclax/tedizolid combination therapy are shown for reference.

The resulting effects on the cellular viability are shown in comparison to the venetoclax/tedizolid combination treatment in Fig 6. Importantly, as illustrated in Fig 6, Mcl-1 inhibition in combination with venetoclax treatment does lead to a substantial decrease in cellular viability compared to venetoclax monotherapy, c.f. Fig 4A. In addition, the changes in the cellular viability closely follow the changes that are observed with the venetoclax/tedizolid combination treatment up until approximately 48 hours after treatment administration. However, after this time, the cellular viability corresponding to venetoclax/Mcl-1 inhibitor combination treatment significantly deviates from the cellular viability observed with venetoclax/tedizolid combination treatment, with the latter resulting in over 40% lower cellular viability on day five of treatment. Since Mcl-1 inhibition in combination with venetoclax treatment is not sufficient to capture the trends in cell viability observed with venetoclax/tedizolid combination therapy, this suggests that there is an additional dominant mechanism by which ISR activation helps to robustly overcome venetoclax resistance.

### 3.3 Temporal sequencing of drugs improves the effectiveness of combination therapy

**Fig 7:**
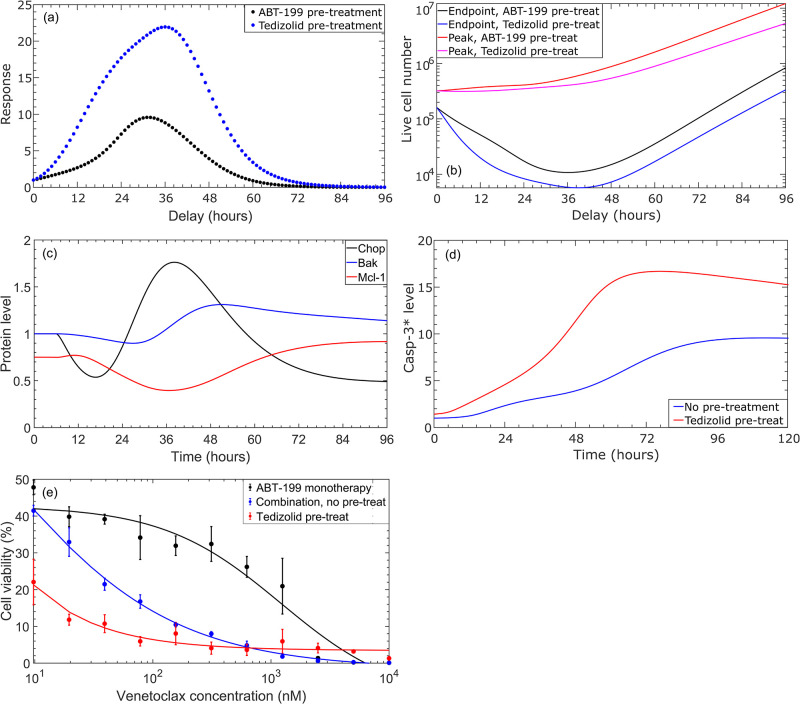
Results of pre-treatment before the administration of 400 nM venetoclax and 5,000 nM tedizolid combination therapy. (a) Simulated response values, calculated using Eq (3), for 400 nM venetoclax administered prior to combination therapy and for 5,000 nM tedizolid administered prior to combination therapy, with the indicated pre-treatment windows. Combination therapy was simulated for 5 days following pre-treatment. (b) Simulated peak and end-point live cell counts during the combination therapy with different pre-treatment windows. In (a) and (b), a pre-treatment window of “0” corresponds to combination therapy without pre-treatment. (c) Simulated expression of Chop, free (inactive) Bak, and free Mcl-1 protein levels after the administration of 5,000 nM tedizolid monotherapy. Each protein was normalized to its total expression in the untreated system. (d) Active Caspase-3 levels, normalized to the level in the untreated system, observed in simulations of combination therapy without pre-treatment and with tedizolid administered 36 hours prior to combination therapy. Time is measured relative to the administration of venetoclax. (e) Experimentally-measured cellular viability for MOLM-13 R2 cells treated with venetoclax monotherapy, combination venetoclax/tedizolid therapy without pre-treatment, or combination therapy with tedizolid pre-treatment administered 48 hours prior, at the indicated venetoclax concentrations. Cellular viability was measured 72 hours after the administration of venetoclax and has been normalized to the cellular viability in the untreated cells. Data points show average and standard deviation of experimental measurements, and solid lines correspond to sigmoidal fits to the data (see text for further details).

The dominant regulatory mechanism by which ISR activation helps to overcome venetoclax resistance became apparent when we explored whether the response to combination therapy could be further improved by temporal sequencing of the drugs. In particular, we simulated pre-treatment with venetoclax prior to administration of combination therapy, as well as tedizolid pre-treatment prior to administration of combination therapy, with pre-treatment windows ranging from one to 96 hours. We calculated the response using Eq (3) for each case, and the results are presented in Fig 7. As shown in Fig 7A, administering venetoclax before combination therapy with a pre-treatment window of up to approximately 60 hours leads to a modest improvement in the effectiveness of the combination therapy, in comparison to combination therapy without pre-treatment. Beyond this point, the response falls below 1 (the value corresponding to combination therapy without pre-treatment), which results from an increase in the peak number of live cells that continues to further grow with increasing pre-treatment window, see Fig 7B. Similar trends are observed for combination therapy with tedizolid pre-treatment, except the improvement in response is much more significant, peaking at approximately 36 hours, see Fig 7A. Importantly, Fig 7B indicates that temporal sequencing of the drugs can significantly reduce the number of live cells at the end of the treatment window, leading to over an order of magnitude reduction in live cells on day 5 of treatment for a 38-hour tedizolid pre-treatment window, compared to combination therapy without pre-treatment.

Since the improvement in treatment response is much more significant with tedizolid pre-treatment, we focused our remaining analysis on this case. To determine the mechanism by which tedizolid pre-treatment is exerting an increased response to combination therapy, we investigated the temporal evolution of protein levels obtained from numerical simulations of tedizolid monotherapy. As illustrated by Fig 7C, the peak in response to combination therapy with pre-treatment correlates strongest with increased Chop expression, decreased free Mcl-1 concentration, and the onset of increased and sustained free Bak concentration, resulting from tedizolid monotherapy. These findings echo the results of Fig 6, in which Mcl-1 inhibition was found to contribute to decreased cellular viability, but did not account for the continuous decrease in cellular viability observed with combination treatment after approximately 48 hours. Our findings indicate that a combination of transient Mcl-1 inhibition and increased Bak production are important mechanisms by which ISR activation helps to overcome venetoclax resistance. Furthermore, the results suggest that administering venetoclax close to the time that free Mcl-1 levels are inhibited and free Bak levels are increased due to tedizolid pre-treatment enables more robust and sustained apoptotic signaling. This is because when Bcl-2 is sequestered from the sensitizer and active effector pro-apoptosis proteins, there will not be enough anti-apoptotic Mcl-1 in the system to subsequently prevent the activation of the pro-apoptosis proteins, especially given the increased expression of Bak.

Indeed, as indicated by Fig 7D, tedizolid pre-treatment leads to a significant increase in the peak active Caspase-3 levels in comparison to combination therapy without pre-treatment. Furthermore, tedizolid pre-treatment leads to a much earlier onset of sufficiently high active Caspase-3 levels, which begins shortly after venetoclax administration, peaks around 72 hours, and is sustained throughout the entire duration of combination therapy. In the combination therapy without pre-treatment, comparable active Caspase-3 levels are not achieved. Furthermore, peak Caspase-3 levels are not reached until close to 96 hours after drug administration, see Fig 7D. Due to the increased and early-onset Caspase-3 activation, tedizolid pre-treatment results in an increase of cumulative active Caspase-3 levels of close to 100% over the treatment window, which leads to significantly more robust apoptotic signaling. Similar trends are observed for active Bax and Bak levels with the delayed combination therapy. Interestingly, while the cell viability was seen to be quite sensitive to small perturbations of the kinetic parameter values, see Fig 5D, the optimal pre-treatment window was robust to changes in the parameter values, with the average and standard deviation being 36.8±1.5 and 37.2±1.4 for perturbations of + 5% and −5%, respectively, to individual kinetic parameter values.

Additional in vitro experiments were conducted to validate the predictions of the mathematical model, namely, that temporal sequencing of the drugs leads to increased treatment efficacy. In Fig 7E we show the results of cellular viability measurements for MOLM-13 R2 cells treated with venetoclax monotherapy, combination treatment with both drugs administered simultaneously, and combination therapy with 48 hours tedizolid pre-treatment. We see from the figure that pre-treatment with tedizolid leads to a significant decrease in cellular viability at most of the venetoclax concentrations in the serial dilution, compared to venetoclax monotherapy and combination therapy without pre-treatment. Unfortunately, the MOLM-13 R2 cells did not maintain their high baseline resistance to venetoclax during the experiments, and thus the cellular viability results are similar for combination therapy with and without pre-treatment at high concentrations of venetoclax. Nevertheless, the data do clearly indicate that pre-treatment with tedizolid leads to a significant and increased sensitization to venetoclax treatment, confirming the predictions of the mathematical model. We note that the solid lines in Fig 7E correspond to sigmoidal fits with the form *y* = *a* + (*b*−*a*)*x*^*n*^/(*x*^*n*^ + *K*^*n*^), where (*a*, *b*, *K*, *n*) = (−0.1033, 0.4281, 1218, −0.8648)) for venetoclax monotherapy, (−0.01526, 1.088, 4.636, −0.5862) for combination therapy without tedizolid pre-treatment, and (0.0343, 87.47, 0.003124, −0.7694) for combination therapy with tedizolid pre-treatment.

## 4 Discussion

In this work, we developed a multi-scale systems biology approach to study the mechanisms by which ISR activation helps to overcome venetoclax resistance in acute myeloid leukemia. The model consists of a module for drug uptake, the regulation of transcription factor expression, the regulation of Bcl-2 family proteins by the transcription factors c-Myc and Chop, Bcl-2 family protein interactions, direct and indirect drug effects on the Bcl-2 family proteins and the cellular metabolism, and cellular proliferation and death. This multi-scale model enables the integration of RNA-level, protein-level, and cellular viability and proliferation data (see also Ref. [116]). Specifically, the approach captures the experimentally-observed trends in protein expression levels and cellular viability and proliferation for all treatment protocols (venetoclax monotherapy, tedizolid monotherapy, and venetoclax/tedizolid combination therapy), see Figs 3 and 4.

Interestingly, the kinetic parameters obtained by fitting to the experimental data correspond to regulatory functions that are consistent with the hormetic-like response that has been previously hypothesized for c-Myc [131, 132]. Despite not imposing hard concentration cutoffs for the rheostat-like regulation of the Bcl-2 family proteins by c-Myc, the predicted regulatory functions still led to an identifiable “cancer zone”, consisting of a range of c-Myc concentrations wherein the predominant function of c-Myc is to promote cell growth, as well as an “apoptosis zone”, wherein the primary function of c-Myc is to promote cell death, see Fig 3D. We note that during the initial stages of the work, to reduce the number of parameters in the model, we attempted to use a single concentration of c-Myc as the half-saturation constant for all regulatory interactions that promote cell survival (e.g. Bcl-2 upregulation, Mcl-1 upregulation, Bim inhibition), i.e. the onset of the cancer zone, as well as a single concentration for all regulatory interactions that promote apoptosis (e.g. Bcl-2 inhibition, Bax upregulation, Bak upregulation, Bim upregulation), i.e. the onset of the apoptosis zone, see Table A in S1 Text. We call this the “hard cutoff” model because the regulatory nature of c-Myc will switch abruptly at these two concentrations of c-Myc. However, while this led to a reduction in the number of kinetic parameters, this simpler implementation of the mathematical model could not as closely capture the trends in protein expression and cellular viability and proliferation data. The experimental trends were captured much better by the model when the half saturation constants for the individual regulatory events were taken to be unique. This is likely a reflection of the different binding affinities for the different gene promoter regions, as well as the fact that some of the regulatory effects of c-Myc on the Bcl-2 family proteins are non-transcriptional. Thus the model presented in this work, without the hard cutoffs, is more consistent with the underlying biology of the system.

The mathematical model developed in this work surprisingly predicted several important features of the resistant AML cell line that are consistent with experimental data that were not implemented into the fitting procedure. For example, comparison of the resistant versus parental MOLM-13 cell lines indicated that Mcl-1 protein upregulation is a main contributor to venetoclax resistance, with an expression that is over 40 times higher in the resistant cells. In agreement with this finding, local sensitivity analysis identified the expression of Mcl-1 protein as the most sensitive quantity in the untreated system. Moreover, Mcl-1 expression was identified to be the most sensitive initial condition with both venetoclax and tedizolid monotherapies, and the second-most sensitive initial condition with the combination therapy, see Fig 5. Local sensitivity analysis also identified Bim protein expression as the second-most sensitive quantity in the untreated system, the second-most sensitive initial condition with venetoclax and tedizolid monotherapies, and the third-most sensitive initial condition with combination therapy, see Fig 5. Experimentally, Bim expression was found to be nearly three times higher in the resistant cell line compared to the parental cell line. Moreover, these findings agree with previous work [165] that showed that cellular addiction to pro-survival Bcl-2 family proteins follows when cells are “primed for death” by the expression of activator BH3-only proteins. This “priming” with pro-death signals leads to a significantly different physiology than what is observed in healthy cells, leaving the latter selectively vulnerable to treatment.

During the fitting procedure, a subset of the half-saturation constants tended toward large values, so we increased the upper bound of the search space for these parameters if required. The result is that a subset of the regulatory interactions observed previously in the literature were found not to significantly contribute to the treatment response in the MOLM-13 R2 cell line. This includes the inhibition of Bcl-2 expression by Chop and the upregulation of Bax expression by Chop. For these regulatory interactions, c-Myc was found to be a stronger contributor to the regulation of Bcl-2 and Bax protein expression. This could be an artifact of the simplicity of the model, or it could reflect the significant regulatory role of c-Myc in the apoptosis pathway in MOLM-13 R2 cells, which is not surprising since c-Myc is elevated and promotes tumorigenesis in a majority of human cancers [117–121]. As indicated by Fig 7C, the predominant mechanisms by which Chop contributes to the treatment response is through the upregulation of Bak expression and the inhibition of Mcl-1 expression. Interestingly, the c-Myc-dependent contribution to the cellular proliferation, see Eq. (A.14) in S1 Text, was found to be minimal. Evidently, the suppression of cellular respiration and glycolysis observed with the administration of venetoclax and tedizolid [26] were effectively captured by the remaining terms in Eq. (A.14). Significantly, we see from Figs 3 and 4 that the effectiveness of the combination treatment does not rely on substantial and sustained changes to the expression of the Bcl-2 family proteins [26]. Rather, as indicated by the results of the timing experiments, transient but concerted changes of small magnitude in the expression profiles of the Bcl-2 family proteins are sufficient to induce sustained apoptotic signalling and heightened response to combination treatment, see Fig 7. This leads us to the conclusion that it is the robust apoptotic signalling resulting from *transient* changes to the expression of the Bcl-2 family proteins, combined with the effective *lowering of the apoptotic threshold* due to the suppression of cellular metabolism, that underlies the most effective treatment strategy for overcoming resistance to venetoclax.

In conclusion, the integrative systems biology approach developed in this work led to the identification of the most significant mechanisms of ISR activation that help to overcome venetoclax resistance in acute myeloid leukemia, as well as the determination of the optimal combination treatment protocol. Further work is required to investigate how the results extend to AML cell lines that depend on Bcl-XL upregulation as a primary mechanism to acquired venetoclax resistance, as well as to in vivo settings. The effective translation of the results to clinical practice is impeded by the limitations of the model, which include a large number of free parameters and several simplifying assumptions. However, extensions to the model may help in this endeavour, such as the inclusion of in vivo data into the fitting procedure, expanding the model to include additional Bcl-2 family proteins (subject to data availability), and implementing different cell phenotypes (stem versus non-stem cells) [26]. In addition, simulated clinical trials, generated using the model, can be integrated with machine learning methods using transfer learning, see e.g. Ref. [166], offering the potential to significantly improve the predictive power of these algorithms for real patient data. Finally, while this study was focused on acquired resistance to venetoclax, implementing cellular heterogeneity into the model to include a population of cells that are sensitive and a population of cells that are resistant to venetoclax could give insight into how to overcome intrinsic drug resistance in AML.

It would also be interesting to develop a more sophisticated cell growth model that accurately captures the distinct changes in cellular respiration, glycolysis, and reactive oxygen species production resulting from drug administration. Such a growth model could be parameterized by available metabolic and glycolytic data [26]. Finally, it would be worth considering how the results are affected by stochasticity in the system. For example, the binding affinities between the Bcl-2 family proteins may not be constant due to environmental fluctuations in polar molecules, electric fields, etc., and the production rates of the Bcl-2 family proteins could fluctuate depending on, for example, ribosome and amino acid availability. In future work, we will implement these extensions to the model to improve the clinical translatability of the approach, thus helping to improve patient prognosis for those suffering with AML.

## Supporting information

S1 Text Supplementary Information file. The file contains Appendices A, B, and C, which include all of the equations for the mathematical model, tables of the individual molecular interactions and parameter values, and the results of global sensitivity analysis.
(PDF)
