# Supplementary material for: An integrative systems biology approach to overcome venetoclax resistance in acute myeloid leukemia
Source: PLoS Comput Biol. 2022 Sep 13;18(9):e1010439. doi: 10.1371/journal.pcbi.1010439 (PMC9469948; doi:10.1371/journal.pcbi.1010439)
Supplement: S1 Text — The file contains Appendices A, B, and C, which include all of the equations for the mathematical model, tables of the individual molecular interactions and parameter values, and the results of global sensitivity analysis. (PDF) [file pcbi.1010439.s001.pdf]

An integrative systems biology approach to overcome venetoclax resistance in acute myeloid leukemia

Michelle Przedborski[1], David Sharon[2], Severine Cathelin[2], Steven Chan[2], and Mohammad Kohandel[1]

[1]Department of Applied Mathematics, University of Waterloo, Waterloo, ON, Canada
[2]Princess Margaret Cancer Centre, Toronto, ON, Canada

August 5, 2022

Supplementary Information

A Mathematical Model

The mathematical model developed in this work consists of several modules, from cellular drug uptake, to drug effects on transcription factor expression, to the regulatory effects of transcription factors on the Bcl-2 family protein levels, to the interactions between the Bcl-2 family proteins, to the activation of Caspase-3 (signalling the onset of apoptosis), to feedback of Caspase-3 on the Bcl-2 family proteins. We also develop a simplistic model to capture the metabolic effects of the drugs and their subsequent impact on cellular proliferation, as well as the initiation of cell death by active Caspase-3. Here we describe the equations that model each of these processes. In the next section, we present the parameter values that were measured experimentally and were available in the literature, as well as parameter values that were obtained in this work by fitting to the experimental data.

A.1 Drug uptake and regulation of transcription factors

The first two modules, cellular drug uptake and the drug effects on transcription factor expression, were modeled as described in Ref. [1]. Here we give a brief overview of the model and refer the reader to Ref. [1] for more details. In this approach, the in vitro cellular uptake and decay of drug $D = \{A, T\}$ for venetoclax or tedizolid, respectively, was modelled as a system of two coupled ordinary differential equations (ODEs) [1]:

$$\begin{aligned} \frac{d[D_e]}{dt} &= -\delta_{D_e}[D_e] \\ \frac{d[D_i]}{dt} &= k_D[D_e] - \delta_{D_i}[D_i], \end{aligned} \tag{A.1}$$

where t is the time, $[D_e] \equiv [D_e(t)]$ denotes the extra-cellular concentration of drug D, and $[D_i] \equiv [D_i(t)]$ is the intra-cellular concentration. In numerical simulations, it is assumed that, initially, $[D_e(t = 0)]$ is equal the administered dose and $[D_i(t = 0)] = 0$.

The mean-field model with temporal delay [1] was utilized in this work to model the expression of c-Myc and Chop in the presence of drug administration. Following this convention, we will refer to the transcription factors c-Myc and Chop as "target proteins", and their concentration as $[T_j]$. In this approach, the temporal evolution of $[T_j]$ are modeled by treating the *drugs as transcriptional regulators*, assuming the quasi-steady state for mRNA expression levels [2]. The monotherapy with either ABT-199 or Tedizolid perturbs the regulatory networks of the target proteins, resulting in four distinct possible effects on the target protein expression, which are: (1) increase in production rate, (2) decrease in production rate, (3) increase in stability, and (4) decrease in stability. Rather than distinguishing between the underlying mechanisms leading to each drug effect, an appropriate (mean) contribution is included in the model to capture the overall regulatory behaviour. For a

single therapeutic agent, the time evolution of $[T_j]$ is given by the following first-order ODE [1]:

$$\frac{d[T_j]}{dt} = k_{T_j,0}\Big(1 + k_{T_j,1} H^{(1)}_{j,m,1}\Big) H^{(2)}_{j,m,2} - \Big(\delta_{T_j,0} - \big[\delta_{T_j,0} - \delta_{T_j,\min}\big] H^{(1)}_{j,m,3} - \big[\delta_{T_j,0} - \delta_{T_j,\max}\big] H^{(1)}_{j,m,4}\Big)[T_j]. \tag{A.2}$$

In the above equation $j = 1, 2$ corresponds to c-Myc or Chop, respectively, subscript m labels the drug ($m = 1, 2$ corresponds to venetoclax or tedizolid, respectively), $k_{T_j,0}$ is the background production rate for target protein T_j in the untreated case, $k_{T_j,1}$ is a multiplier that dictates the maximum production rate for T_j in the presence of drug, and $\delta_{T_j,0}$, $\delta_{T_j,\min}$, and $\delta_{T_j,\max}$ are, respectively, the nominal, minimum and maximum decay rates for T_j. We note that $k_{T_j,0}$, $k_{T_j,1}$, $\delta_{T_j,0}$, $\delta_{T_j,\min}$, and $\delta_{T_j,\max}$ are biologically-constrained parameters that do not depend on the drug concentration. The drug concentration is contained in the terms $H^{(p)}_{j,m,\ell}$, where $p = 1, 2$, which are Hill functions [2–4] defined by:

$$H^{(1)}_{j,m,\ell} = \frac{[X_{j,m,\ell}]^{n_{j,m,\ell}}}{1 + [X_{j,m,\ell}]^{n_{j,m,\ell}}}, \tag{A.3}$$

and

$$H^{(2)}_{j,m,\ell} = 1 - H^{(1)}_{j,m,\ell} = \frac{1}{1 + [X_{j,m,\ell}]^{n_{j,m,\ell}}}. \tag{A.4}$$

In Eqs. (A.3) and (A.4),

$$[X_{j,m,\ell}] = \frac{[D_{m,i}]}{K_{j,m,\ell}} \tag{A.5}$$

is the time-dependent intra-cellular drug concentration for drug D_m (as indicated by the additional subscript "i" in $[D_{m,i}]$, see Eq. (A.1) normalized by the half-saturation constant $K_{j,m,\ell}$ (where $K_{j,m,\ell} > 0$) for target protein T_j and effect ℓ, where $\ell = 1, \ldots, 4$. In the absence of drugs, Eq. (A.2) reaches a steady state at sufficiently long times, where $[T_j]$ saturates at the value of $k_{T_j,0}/\delta_{T_j,0}$. This is taken to be the initial condition for the treated system in numerical simulations.

Following Ref. [1], the effects of combination therapy on the time evolution of $[T_j]$ is described by the ODE:

$$\frac{d[T_j]}{dt} = k_{T_j,0}\Big(1 + k_{T_j,1} \mathcal{H}^{(1)}_{j,1}\Big) \mathcal{H}^{(2)}_{j,2} - \Big(\delta_{T_j,0} - \big[\delta_{T_j,0} - \delta_{T_j,\min}\big] \mathcal{H}^{(1)}_{j,3} - \big[\delta_{T_j,0} - \delta_{T_j,\max}\big] \mathcal{H}^{(1)}_{j,4}\Big)[T_j]. \tag{A.6}$$

The form of this equation is identical to Eq. (A.2), but with the replacement of the standard Hill functions $H^{(p)}_{j,m,\ell}$ with the extended Hill functions $\mathcal{H}^{(1)}_{j,\ell}$ and $\mathcal{H}^{(2)}_{j,\ell}$, which are defined by:

$$\mathcal{H}^{(1)}_{j,\ell} = \frac{C_{j,\ell}}{1 + C_{j,\ell}} \tag{A.7}$$

and

$$\mathcal{H}^{(2)}_{j,\ell} = 1 - \mathcal{H}^{(1)}_{j,\ell} = \frac{1}{1 + C_{j,\ell}}, \tag{A.8}$$

where

$$C_{j,\ell} = \sum_{m=1}^{2} w_{j,m,\ell}[X_{j,m,\ell}]^{n_{j,m,\ell}} + q_{j,\ell} \prod_{m=1}^{2} w_{j,m,\ell}[X_{j,m,\ell}]^{n_{j,m,\ell}}. \tag{A.9}$$

It is clear that $\mathcal{H}^{(1)}_{j,\ell}$ and $\mathcal{H}^{(2)}_{j,\ell}$ are Hill functions of the argument $C_{j,\ell}$, which as Eq. (A.9) illustrates, contains all instances of the drug concentrations, $[X_{j,m,\ell}]$. Following the work of Ref. [5], the weights $w_{j,m,\ell}$, where $w_{j,m,\ell} \geq 0 \; \forall \; \{j,\, m,\, \ell\}$, were introduced to incorporate antagonistic drug effects. The weights distinguish the relative importance of the individual drug effect to the combined effect during combination therapy: taking $w_{j,m,\ell} = 1$ corresponds to each drug contributing equally to the combined effect (the default value), while taking $w_{j,m,\ell} \approx 0$ captures cases where combined antagonistic drug effects nullify or dampen a pathway that plays a key role in the individual response to one or both drugs. Importantly, taking $w_{j,m,\ell} = 1$ in Eq. (A.9) gives the familiar expression for protein regulation by two transcription factors, where cooperativity between transcription factor binding is described by the constant factor $q_{j,\ell}$ [2,5]. This cooperativity between

two or more drugs might arise when the protein networks affected by each drug overlap or have synergistic physiological functions [1].

Since the mean-field approach does not model individual molecular pathways, temporal delays can be incorporated into the drug effects to account for regulatory interactions that are downstream of several other protein interactions. For example, if the administration of a drug affects the expression of a protein that is upstream of a regulatory pathway, there may be a series of interactions, including transcription and translation events and post-translational modifications, that must occur before the regulatory protein is affected. In this scenario, there should be a time delay between drug administration and the corresponding effect on the target protein. To incorporate temporal delays into the drug effects, we make the replacement $[X_{j,m,\ell}](t) = [D_{m,i}]\big(t - \tau_{j,m,\ell}\big)/K_{j,m,\ell}$, where $\tau_{j,m,\ell}$ is the time delay. The kinetic parameter values that were previously determined for the MOLM-13 R2 cell line using the mean-field approach with temporal delay are presented in Table D.

A.2 Regulation of Bcl-2 family proteins by transcription factors

In Table A, we present the direct and indirect individual regulatory effects of the transcription factors c-Myc and Chop on the expression of the Bcl-2 family protein levels that were included in the model. Square brackets denote the concentration of the corresponding protein, t denotes the time, and $\frac{d[X]}{dt}$ denotes the time-derivative of the concentration of protein X. In addition, $k_{0,X}$, k_X, n_i, and K_i are constants. The constant $k_{0,X}$ represents the background (basal) production rate for protein X, and the maximal production rate is given by $k_{0,X} + k_X$. We use Hill functions [3, 4] to model the regulatory effects. Furthermore, we make the quasi-steady state approximation for mRNA levels so that protein production can be treated as a reduced one-step process [2]. The shape and steepness of the Hill function for regulatory effect i is determined by the value of the exponent n_i, which is referred to as the Hill coefficient due to its origin in the context of ligand binding to macro-molecules [3]. The constant K_i is the half-saturating concentration for the transcription factor in the regulatory event i. Further details about the regulatory interactions are described in Section 2.1.3 of the manuscript for c-Myc and Section 2.1.4 for Chop.

As indicated in Table A, each of the Bcl-2 protein family members is regulated by both c-Myc and Chop. Furthermore, due to the bistable response of c-Myc [8, 21], some of the Bcl-2 protein family members experience two different regulatory mechanisms due to c-Myc alone, depending on the c-Myc concentration. When multiple transcription factors regulate the expression of a gene, their effects must be considered simultaneously. To model the combined regulatory effects of c-Myc and Chop, including the bistable response of c-Myc [8, 21], we follow the work of Refs. [2, 5]. The only exception to this is for the combined regulatory effects of Bim since the inhibitory effect of c-Myc on Bim expression is the prevention of Bim accumulation in the mitochondria, see Section 2.1.3 of the manuscriptand Refs. [6, 9, 10]. Thus, even if Chop concentration is sufficient to induce Bim expression, it will not accumulate in the mitochondria to play a role in the apoptosis pathway, and this is taken into consideration in the ODE for Bim, c.f. Eqs. (A.20) and (A.21) and their following descriptions, which are presented in Section A.7, after first discussing the additional components of the model.

We note that in implementing the convention in Refs. [2, 5] for the combined regulatory effects of multiple transcription factors, we make the following assumptions. (i) Each Bcl-2 family member has a promoter with at least two non-overlapping operator regions, and each transcription factor can bind to its own operator region to regulate the expression of a target gene. (ii) The rate of gene expression contains a constant background (basal) production term that is independent of the concentration of the transcription factors, denoted by $k_{0,X}$ for protein X. (iii) Following the hypothesized bistable response of c-Myc [8, 21], c-Myc is an activator of gene expression for Mcl-1 and Bcl-2 at lower concentrations and a repressor at higher concentrations; similarly, c-Myc is a repressor of gene expression for Bim at lower concentrations and an activator of Bim, Bax, and Bak expression at higher concentrations. This amounts to assuming that $K_2 < K_4$ and $K_3 < K_5$. We do not impose additional constraints on the half-saturation constants or define a hard cut-off that segregates the hypothesized cancer zone and apoptosis zone. (iv) If a repressor is bound to its operator region, activated gene expression is fully blocked, except for the basal expression level.

Finally, we point out that our model neglects the inhibitory effects of ISR activation on protein translation rates. We justify this assumption by noting that, under experimental conditions, EIF2-α is dephosphorylated by GADD34 within hours of ISR activation [22], thus global translational

Table A: Individual regulatory effects of the transcription factors c-Myc and Chop on the Bcl-2 family proteins.

Description	Rate law	Reference
c-Myc transcriptionally activates Mcl-1	$\frac{d[\text{Mcl-1}]}{dt} = k_{0,\text{Mcl-1}} + k_{\text{Mcl-1}} \frac{[\text{c-Myc}]^{n_1}}{[\text{c-Myc}]^{n_1} + K_1^{n_1}}$	[6]
c-Myc transcriptionally activates Bag-1, upregulating the pro-survival function of Bcl-2	$\frac{d[\text{Bcl-2}]}{dt} = k_{0,\text{Bcl-2}} + k_{\text{Bcl-2}} \frac{[\text{c-Myc}]^{n_2}}{[\text{c-Myc}]^{n_2} + K_2^{n_2}}$	[7,8]
c-Myc transcriptionally represses GADD45a, preventing Bim translocation to the mitochondria	$\frac{d[\text{Bim}]}{dt} = k_{0,\text{Bim}} + k_{\text{Bim}} \frac{K_3^{n_3}}{[\text{c-Myc}]^{n_3} + K_3^{n_3}}$	[9,10]
c-Myc transcriptionally represses Bcl-2	$\frac{d[\text{Bcl-2}]}{dt} = k_{0,\text{Bcl-2}} + k_{\text{Bcl-2}} \frac{K_4^{n_4}}{[\text{c-Myc}]^{n_4} + K_4^{n_4}}$	[8,11–16]
c-Myc transcriptionally activates Bim	$\frac{d[\text{Bim}]}{dt} = k_{0,\text{Bim}} + k_{\text{Bim}} \frac{[\text{c-Myc}]^{n_5}}{[\text{c-Myc}]^{n_5} + K_5^{n_5}}$	[8,11–16]
c-Myc transcriptionally activates Bax	$\frac{d[\text{Bax}]}{dt} = k_{0,\text{Bax}} + k_{\text{Bax}} \frac{[\text{c-Myc}]^{n_6}}{[\text{c-Myc}]^{n_6} + K_6^{n_6}}$	[8,11–16]
c-Myc transcriptionally activates Bak	$\frac{d[\text{Bak}]}{dt} = k_{0,\text{Bak}} + k_{\text{Bak}} \frac{[\text{c-Myc}]^{n_7}}{[\text{c-Myc}]^{n_7} + K_7^{n_7}}$	[8,11–16]
Chop transcriptionally represses Mcl-1	$\frac{d[\text{Mcl-1}]}{dt} = k_{0,\text{Mcl-1}} + k_{\text{Mcl-1}} \frac{K_8^{n_8}}{[\text{Chop}]^{n_8} + K_8^{n_8}}$	[17]
Chop transcriptionally represses Bcl-2	$\frac{d[\text{Bcl-2}]}{dt} = k_{0,\text{Bcl-2}} + k_{\text{Bcl-2}} \frac{K_9^{n_9}}{[\text{Chop}]^{n_9} + K_9^{n_9}}$	[17–19]
Chop transcriptionally activates Bim	$\frac{d[\text{Bim}]}{dt} = k_{0,\text{Bim}} + k_{\text{Bim}} \frac{[\text{Chop}]^{n_{10}}}{[\text{Chop}]^{n_{10}} + K_{10}^{n_{10}}}$	[17–19]
Chop upregulates Bax via transcriptional activation and indirect mechanisms	$\frac{d[\text{Bax}]}{dt} = k_{0,\text{Bax}} + k_{\text{Bax}} \frac{[\text{Chop}]^{n_{11}}}{[\text{Chop}]^{n_{11}} + K_{11}^{n_{11}}}$	[18,20]
Chop transcriptionally activates Bak	$\frac{d[\text{Bak}]}{dt} = k_{0,\text{Bak}} + k_{\text{Bak}} \frac{[\text{Chop}]^{n_{12}}}{[\text{Chop}]^{n_{12}} + K_{12}^{n_{12}}}$	[19]

inhibition occurs over a short time scale. In comparison, the experiments considered in this work span several days, and we therefore expect the inhibitory effects of EIF2-α phosphorylation on the expression of the proteins in our model to be minimal over the long-term. As discussed in Section 2.1.4 of the manuscript, it has recently been proposed that there are two nodes for translational inhibition in the ISR. In particular, in a delayed response to prolonged conditions of cellular stress, transcriptional and post-translational regulatory effects shift the cellular response toward cap-independent translation [22]. Importantly, this results in the selective translation of stress and apoptosis proteins, in addition to proteins required for regulatory cell processes, such as c-Myc [22–24]. While Mcl-1 was seen to be downregulated when cap-dependent translation was inhibited in human leukemia cells [25], we note that this effect is implicitly captured in the model by the inhibitory effect of Chop on Mcl-1 expression. Importantly, these simplifications and assumptions are consistent with the long-term effects of ISR activation and also prevent the unnecessary introduction of additional kinetic parameters into the model.

A.3 Bcl-2 family protein interactions

We limit the scope of the model to the Bcl-2 family proteins for which treated experimental expression levels were available. Furthermore, as noted in Section 2.3 of the manuscript, we excluded certain Bcl-2 family proteins from the model based on redundancy in protein function and also based on their lower expression in the resistant cell line, indicating a limited role in the development of resistance to venetoclax. This enabled a significant decrease in the number of kinetic

parameters in the model, while still retaining sufficient detail to capture the main interactions in the system. Specifically, the model includes the expression of, and interactions between, Bcl-2, Mcl-1, Bim, Bax, and Bak. The interactions, including the reaction equations and corresponding rate laws, are presented in Table B. We note that protein complexes are indicated by the notation $[X{:}Y]$, and k_i, k_{-i}, and κ_i are constants which denote, respectively, association rates, dissociation rates, and activation rates.

Table B: Bcl-2 family protein-protein interactions comprising the apoptosis pathway in the model.

Description	Reaction equation	Rate laws	Reference
Mcl-1 binds to and dissociates from Bim	$\text{Mcl-1} + \text{Bim} \underset{k_{-1}}{\overset{k_1}{\rightleftharpoons}} \text{Mcl-1:Bim}$	$\frac{d[\text{Mcl-1}]}{dt} = \frac{d[\text{Bim}]}{dt} = -\frac{d[\text{Mcl-1:Bim}]}{dt}$ $= -k_1[\text{Mcl-1}][\text{Bim}] + k_{-1}[\text{Mcl-1:Bim}]$	[26–30]
Bcl-2 binds to and dissociates from Bim	$\text{Bcl-2} + \text{Bim} \underset{k_{-2}}{\overset{k_2}{\rightleftharpoons}} \text{Bcl-2:Bim}$	$\frac{d[\text{Bcl-2}]}{dt} = \frac{d[\text{Bim}]}{dt} = -\frac{d[\text{Bcl-2:Bim}]}{dt}$ $= -k_2[\text{Bcl-2}][\text{Bim}] + k_{-2}[\text{Bcl-2:Bim}]$	[26–30]
Bim binds to and activates Bax	$\text{Bim} + \text{Bax} \underset{k_{-3}}{\overset{k_3}{\rightleftharpoons}} \text{Bim:Bax} \xrightarrow{\kappa_3} \text{Bim} + \text{Bax*}$	$\frac{d[\text{Bax}]}{dt} = -k_3[\text{Bim}][\text{Bax}] + k_{-3}[\text{Bim:Bax}]$ $\frac{d[\text{Bim}]}{dt} = -\frac{d[\text{Bim:Bax}]}{dt} = -k_3[\text{Bim}][\text{Bax}]$ $+k_{-3}[\text{Bim:Bax}] + \kappa_3[\text{Bim:Bax}]$ $\frac{d[\text{Bax*}]}{dt} = \kappa_3[\text{Bim:Bax}]$	[26, 27, 29–32]
Bim binds to and activates Bak	$\text{Bim} + \text{Bak} \underset{k_{-4}}{\overset{k_4}{\rightleftharpoons}} \text{Bim:Bak} \xrightarrow{\kappa_4} \text{Bim} + \text{Bak*}$	$\frac{d[\text{Bak}]}{dt} = -k_4[\text{Bim}][\text{Bak}] + k_{-4}[\text{Bim:Bak}]$ $\frac{d[\text{Bim}]}{dt} = -\frac{d[\text{Bim:Bak}]}{dt} = -k_4[\text{Bim}][\text{Bak}]$ $+k_{-4}[\text{Bim:Bak}] + \kappa_4[\text{Bim:Bak}]$ $\frac{d[\text{Bak*}]}{dt} = \kappa_4[\text{Bim:Bak}]$	[26, 27, 29–33]
Mcl-1 binds to and inhibits activated Bax	$\text{Mcl-1} + \text{Bax*} \underset{k_{-5}}{\overset{k_5}{\rightleftharpoons}} \text{Mcl-1:Bax*}$	$\frac{d[\text{Mcl-1}]}{dt} = \frac{d[\text{Bax*}]}{dt} = -\frac{d[\text{Mcl-1:Bax*}]}{dt}$ $= -k_5[\text{Mcl-1}][\text{Bax*}] + k_{-5}[\text{Mcl-1:Bax*}]$	[26, 27, 29, 30, 34]
Mcl-1 binds to and inhibits activated Bak	$\text{Mcl-1} + \text{Bak*} \underset{k_{-6}}{\overset{k_6}{\rightleftharpoons}} \text{Mcl-1:Bak*}$	$\frac{d[\text{Mcl-1}]}{dt} = \frac{d[\text{Bak*}]}{dt} = -\frac{d[\text{Mcl-1:Bak*}]}{dt}$ $= -k_6[\text{Mcl-1}][\text{Bak*}] + k_{-6}[\text{Mcl-1:Bak*}]$	[26, 27, 29, 30, 35, 36]
Bcl-2 binds to and inhibits activated Bax	$\text{Bcl-2} + \text{Bax*} \underset{k_{-7}}{\overset{k_7}{\rightleftharpoons}} \text{Bcl-2:Bax*}$	$\frac{d[\text{Bcl-2}]}{dt} = \frac{d[\text{Bax*}]}{dt} = -\frac{d[\text{Bcl-2:Bax*}]}{dt}$ $= -k_7[\text{Bcl-2}][\text{Bax*}] + k_{-7}[\text{Bcl-2:Bax*}]$	[26, 27, 29, 30, 37]
Bcl-2 binds to and inhibits activated Bak	$\text{Bcl-2} + \text{Bak*} \underset{k_{-8}}{\overset{k_8}{\rightleftharpoons}} \text{Bcl-2:Bak*}$	$\frac{d[\text{Bcl-2}]}{dt} = \frac{d[\text{Bak*}]}{dt} = -\frac{d[\text{Bcl-2:Bak*}]}{dt}$ $= -k_8[\text{Bcl-2}][\text{Bak*}] + k_{-8}[\text{Bcl-2:Bak*}]$	[26, 27, 29, 30, 35]

A.4 Caspase activation and feedback

As discussed in Section 2.1.1 of the manuscript, the activated pro-apoptosis effector proteins Bax and Bak form homo- and heterodimers and higher order oligomers which embed into the mitochondrial outer membrane (MOM). This promotes mitochondrial outer membrane permeabilization (MOMP) and the release of cytochrome C into the cytosol, which is followed by the formation of the apoptosome, the activation of several Caspases and subsequent negative feedback on the pro-survival Bcl-2 family proteins, and ultimately cell death. To model these processes, we use a simplistic approach that enables a reduction in the number of equations and kinetic parameters in the mathematical model. Specifically, we do not directly model the formation of active Bax and Bak homo- and heterodimers and oligomers, the process of MOMP, or the Caspase cascade. Rather, as indicated in Table C, we take the level of active Caspase-3 to be indicative of the onset of apoptosis since Caspase-3 is known to be an executioner of apoptosis. Furthermore, we make the simplifying assumption that the level of active Caspase-3 depends directly on the concentrations of active Bax and Bak in the system. Finally, we include the negative feedback of Caspase-3 on the

pro-survival Bcl-2 family proteins, which amplifies the apoptotic signalling at the mitochondria.

Table C: Caspase interactions in the apoptosis pathway that are included in the mathematical model.

Description	Reaction equation	Rate laws	Reference
Activated Bax promotes MOMP and caspase activation	$\text{Bax}^* + \text{Casp-3} \xrightarrow{k_9} \text{Bax}^* + \text{Casp-3}^*$	$\frac{d[\text{Casp-3}]}{dt} = -\frac{d[\text{Casp-3}^*]}{dt} = -k_9[\text{Bax}^*][\text{Casp-3}]$	[38–46]
Activated Bak promotes MOMP and caspase activation	$\text{Bak}^* + \text{Casp-3} \xrightarrow{k_{10}} \text{Bak}^* + \text{Casp-3}^*$	$\frac{d[\text{Casp-3}]}{dt} = -\frac{d[\text{Casp-3}^*]}{dt} = -k_{10}[\text{Bak}^*][\text{Casp-3}]$	[38–40, 43–46]
Activated caspase 3 cleaves and inhibits Mcl-1	$\text{Casp-3}^* + \text{Mcl-1} \xrightarrow{k_{11}} \text{Casp-3}^* + \text{Mcl-1}^-$	$\frac{d[\text{Mcl-1}]}{dt} = -\frac{d[\text{Mcl-1}^-]}{dt} = -k_{11}[\text{Mcl-1}][\text{Casp-3}^*]$	[47]
Activated caspase 3 cleaves and inhibits Bcl-2	$\text{Casp-3}^* + \text{Bcl-2} \xrightarrow{k_{12}} \text{Casp-3}^* + \text{Bcl-2}^-$	$\frac{d[\text{Bcl-2}]}{dt} = -\frac{d[\text{Bcl-2}^-]}{dt} = -k_{12}[\text{Bcl-2}][\text{Casp-3}^*]$	[48]

A.5 Interaction of venetoclax with Bcl-2 family proteins

Venetoclax (ABT-199) is a potent and specific inhibitor of the Bcl-2 protein. It works by sequestering and binding to Bcl-2 and forming a protein-drug complex with a high binding affinity. This prevents Bcl-2 from carrying out its anti-apoptotic functions, specifically, from binding to the BH3-only proteins and the pro-apoptosis effector proteins. We model this interaction as follows:

$$\text{Bcl-2} + \text{ABT-199} \underset{k_{-13}}{\overset{k_{13}}{\rightleftharpoons}} \text{Bcl-2:ABT-199}, \tag{A.10}$$

where k_{13} and k_{-13} are, respectively, the association and dissociation rates. The corresponding rate laws for this interaction are given by:

$$\frac{d[\text{Bcl-2}]}{dt} = \frac{d[\text{ABT-199}]}{dt} = -\frac{d[\text{Bcl-2:ABT-199}]}{dt} = -k_{13}[\text{Bcl-2}][\text{ABT-199}] + k_{-13}[\text{Bcl-2:ABT-199}], \tag{A.11}$$

where [ABT-199] denotes the intracellular concentration of venetoclax that is not bound to Bcl-2.

In addition to the regulatory effects induced by c-Myc and Chop that resulted from the administration of venetoclax, the protein expression data indicated that venetoclax treatment causes a significant decrease in the expression of Bax. Importantly, this downregulation in Bax expression could not be explained by the direct and indirect regulatory effects of c-Myc and Chop outlined in Table A. This is because on day 3 of ABT-199 treatment, c-Myc expression has increased, see Figure 3 of the manuscript. Since c-Myc upregulates Bax expression, we would expect to see a resulting *increase* in Bax levels during treatment. On the other hand, if a decrease in Chop expression were the cause of the drop in Bax levels on day 3 of ABT-199 treatment, then the same mechanism would cause a decrease in Bax levels with tedizolid treatment as well, which is inconsistent with experimental measurements, see Figure 3 of the manuscript. Consequently, there is another mechanism that is leading to the marked decrease in Bax expression during ABT-199 treatment.

As discussed in Section 2.1.2, activation of the PI3K/AKT pathway has been commonly observed in cells after both short and long-term exposure to venetoclax treatment [49]. Furthermore, it has been previously reported [50] that AKT phosphorylates Bax at residue S184, which impedes its ability oligomerize and function as a pro-apoptosis protein and also significantly decreases its protein stability [51, 52]. Since these changes provided biological rationale for the downregulation of Bax expression with venetoclax treatment, we included an inhibitory term in the model which depends on the concentration of venetoclax. We point out that this inhibitory term was taken to have the form of a Hill-function and was applied to the production rate of Bax to capture the

collective changes of Bax phosphorylation:

$$\frac{d[\text{Bax}]}{dt} = k_{0,\text{Bax}} + k_{\text{Bax}} \frac{K_{13}^{n_{13}}}{[\text{ABT-199}_{\text{total}}]^{n_{13}} + K_{13}^{n_{13}}}, \tag{A.12}$$

where K_{13} and n_{13} are constants, and $[\text{ABT-199}_{\text{total}}]$ denotes the total intracellular concentration of venetoclax. Importantly, we include this inhibitory interaction when combining the regulatory effects of the transcription factors on Bax expression in Section A.7. We note that, alternatively, the effects of Bax phosphorylation by AKT could potentially be captured by including a term in the model which decreases the half-life of the Bax protein in a venetoclax-concentration dependent manner. The limitation of this approach is that an additional kinetic parameter must be introduced into the model, namely, the half-life of the phosphorylated protein. Furthermore, this term alone may not collectively capture all of the changes that occur as a result of Bax phosphorylation at S184.

A.6 Cellular proliferation and viability

We use a simplistic approach to model cellular proliferation and cellular death that takes into account the main mechanisms discussed in Sections 2.1.1 - 2.1.4 of the manuscript. Specifically, we assume that the time evolution of the number of live cells in the model can be described by the equation:

$$\frac{dN}{dt} = \big(\alpha - \beta\big)N, \tag{A.13}$$

where $\alpha \equiv \alpha\big([\text{Bcl-2}], [\text{c-Myc}], [\text{Ted}]\big)$ is the rate of cellular proliferation and $\beta \equiv \beta\big([\text{Casp-3}]\big)$ is the rate of cell death. The rate of cellular proliferation depends on several variables to account for the major metabolic effects of the proteins depicted in the pathway in Figure 2 of the manuscript. The functional form for α is taken to be:

$$\alpha = \left(\lambda_0 + \lambda_{\text{Bcl-2}} \frac{[\text{Bcl-2}]^{n_{14}}}{[\text{Bcl-2}]^{n_{14}} + K_{14}^{n_{14}}} + \lambda_{\text{c-Myc}} \frac{[\text{c-Myc}]^{n_{15}}}{[\text{c-Myc}]^{n_{15}} + K_{15}^{n_{15}}}\right) \frac{K_{16}^{n_{16}}}{[\text{Ted}]^{n_{16}} + K_{16}^{n_{16}}}, \tag{A.14}$$

where λ_0, $\lambda_{\text{Bcl-2}}$, $\lambda_{\text{c-Myc}}$, K_{14}, K_{15}, K_{16}, n_{14}, n_{15}, and n_{16} are constants. In Eq. (A.14), the first term describes a constant background production that is independent of the protein expression levels in the model, and the second term describes the stimulatory effect of Bcl-2 on cellular (mitochondrial) respiration [49,53–59], see Section 2.1.1. This term implicitly takes into account the inhibitory effects of venetoclax treatment on cellular respiration since administration of venetoclax causes a significant drop in free Bcl-2 levels. In addition, the cellular proliferation depends on c-Myc concentration to account for the stimulatory effects of c-Myc on glycolysis and cellular respiration [60–69], see Section 2.1.3. Finally, we include an inhibitory term that depends on the intracellular tedizolid concentration, [Ted], to account for the repression of cellular respiration previously observed, see Refs. [59, 70] and Section 2.1.4.

The cellular death rate is taken to depend upon the level of active Caspase-3 [29,30,38,39,71–80], with the following form:

$$\beta = \Omega_{\text{Casp-3}} \frac{[\text{Casp-3*}_{\text{frac}}]^{n_{17}}}{[\text{Casp-3*}_{\text{frac}}]^{n_{17}} + K_{17}^{n_{17}}}, \tag{A.15}$$

where $\Omega_{\text{Casp-3}}$, K_{17} and n_{17} are constants, and $[\text{Casp-3*}_{\text{frac}}]$ is the fraction of total Caspase-3 in the system that is active, i.e. $[\text{Casp-3*}_{\text{frac}}] = [\text{Casp-3*}]/([\text{Casp-3}] + [\text{Casp-3*}])$. While technically not a concentration, we have maintained the square brackets on the quantity for notational convenience.

Using this model, the time evolution of the number of dead cells in the system is described by:

$$\frac{dD}{dt} = \beta N, \tag{A.16}$$

and the cellular viability is calculated as follows:

$$v = \frac{N}{N + D} \times 100\% \tag{A.17}$$

For consistency with experiments, see Section 2.2, in numerical simulations we initialized the number of live cells to 50,000. We initialized the number of dead cells in numerical simulations such that the initial cellular viability was equal to the average of the experimentally measured untreated

cell viability. This amounted to initializing the number of dead cells in numerical simulations to 813, corresponding to an untreated cellular viability of 98.4%. Proceeding this way maintained an approximately constant cellular viability in the untreated simulations, while still capturing the net cellular proliferation observed in experiments. In the case of treatment simulations, drug-induced fluctuations in the protein levels led to time-dependent changes in the cellular proliferation and death rates, and consequently, also in the simulated cellular viabilities.

A.7 System of coupled ODEs for the apoptosis proteins

Now that we have mapped out the individual interactions in the pathway presented in Figure 2, we combine all of the individual rate laws for each apoptosis protein. This leads to a system of coupled ODEs that describe the time evolution of the concentration of each protein species in the network. Below we describe the set of equations.

1. The equation describing the rate of change of free Mcl-1 concentration is given by:

$$\begin{aligned}\frac{d[\text{Mcl-1}]}{dt} = {} & k_{0,\text{Mcl-1}} + k_{\text{Mcl-1}}\left(\frac{\left(\frac{[\text{c-Myc}]}{K_1}\right)^{n_1}}{1+\left(\frac{[\text{c-Myc}]}{K_1}\right)^{n_1}}\right)\left(\frac{1}{1+\left(\frac{[\text{Chop}]}{K_8}\right)^{n_8}}\right) \\ & - k_1[\text{Mcl-1}][\text{Bim}] + k_{-1}[\text{Mcl-1:Bim}] \\ & - k_5[\text{Mcl-1}][\text{Bax*}] + k_{-5}[\text{Mcl-1:Bax*}] \\ & - k_6[\text{Mcl-1}][\text{Bak*}] + k_{-6}[\text{Mcl-1:Bak*}] \\ & - k_{11}[\text{Mcl-1}][\text{Casp-3*}] \\ & - \delta_{\text{Mcl-1}}[\text{Mcl-1}] \qquad (\text{A.18})\end{aligned}$$

The first term is the basal production rate and the second term describes the stimulatory regulatory effect of c-Myc and the inhibitory effect of Chop. The next six terms describe binding and dissociation events with other Bcl-2 family proteins, the seventh term describes cleavage by active Caspase-3, and the last term describes natural protein decay.

2. The equation describing the rate of change of free Bcl-2 concentration is given by:

$$\begin{aligned}\frac{d[\text{Bcl-2}]}{dt} = {} & k_{0,\text{Bcl-2}} + k_{\text{Bcl-2}}\left(\frac{\left(\frac{[\text{c-Myc}]}{K_2}\right)^{n_2}}{1+\left(\frac{[\text{c-Myc}]}{K_2}\right)^{n_2}}\right)\left(\frac{1}{1+\left(\frac{[\text{c-Myc}]}{K_4}\right)^{n_4}}\right)\left(\frac{1}{1+\left(\frac{[\text{Chop}]}{K_9}\right)^{n_9}}\right) \\ & - k_2[\text{Bcl-2}][\text{Bim}] + k_{-2}[\text{Bcl-2:Bim}] \\ & - k_7[\text{Bcl-2}][\text{Bax*}] + k_{-7}[\text{Bcl-2:Bax*}] \\ & - k_8[\text{Bcl-2}][\text{Bak*}] + k_{-8}[\text{Bcl-2:Bak*}] \\ & - k_{12}[\text{Bcl-2}][\text{Casp-3*}] \\ & - k_{13}[\text{Bcl-2}][\text{ABT-199}] + k_{-13}[\text{Bcl-2:ABT-199}] \\ & - \delta_{\text{Bcl-2}}[\text{Bcl-2}] \qquad (\text{A.19})\end{aligned}$$

The first term is the basal production rate and the second term describes the stimulatory regulatory effect of c-Myc at lower concentrations, the inhibitory regulatory effect of c-Myc at higher concentrations ($K_2 < K_4$), and the inhibitory effect of Chop. The next six terms describe binding and dissociation events with other Bcl-2 family proteins, the seventh and eighth terms describe binding and dissociation with venetoclax, the ninth term describes cleavage by active Caspase-3, and the last term describes natural protein decay.

3. The equation describing the rate of change of free Bim concentration is:

$$
\begin{aligned}
\frac{d[\text{Bim}]}{dt} &= k_{0,\text{Bim}} \\
&+ k_{\text{Bim}}\left[\left(1-\frac{\left(\frac{[\text{c-Myc}]}{K_5}\right)^{n_5}}{1+\left(\frac{[\text{c-Myc}]}{K_5}\right)^{n_5}}\right)\left(\frac{\left(\frac{[\text{Chop}]}{K_{10}}\right)^{n_{10}}}{1+\left(\frac{[\text{Chop}]}{K_{10}}\right)^{n_{10}}}\right)\left(\frac{1}{1+\left(\frac{[\text{c-Myc}]}{K_3}\right)^{n_3}}\right)+\frac{\left(\frac{[\text{c-Myc}]}{K_5}\right)^{n_5}}{1+\left(\frac{[\text{c-Myc}]}{K_5}\right)^{n_5}}\right] \\
&- k_1[\text{Mcl-1}][\text{Bim}] + k_{-1}[\text{Mcl-1:Bim}] \\
&- k_2[\text{Bcl-2}][\text{Bim}] + k_{-2}[\text{Bcl-2:Bim}] \\
&- k_3[\text{Bim}][\text{Bax}] + k_{-3}[\text{Bim:Bax}] + \kappa_3[\text{Bim:Bax}] \\
&- k_4[\text{Bim}][\text{Bak}] + k_{-4}[\text{Bim:Bak}] + \kappa_4[\text{Bim:Bak}] \\
&- \delta_{\text{Bim}}[\text{Bim}] \qquad \text{(A.20)}
\end{aligned}
$$

The first term is the basal production rate and the second term describes the inhibitory regulatory effect of c-Myc at lower concentrations, the stimulatory regulatory effect of c-Myc at higher concentrations ($K_3 < K_5$), and the stimulatory effect of Chop. We note that the production terms give dominance to the regulatory effects of c-Myc on the expression of Bim. In particular, the terms in square parentheses capture protein production by c-Myc when [c-Myc] $> K_5$, in which case the stimulatory Hill function will saturate, the maximal production rate $k_{0,\text{Bim}} + k_{\text{Bim}}$ is achieved, and production by Chop is redundant. For lower concentrations of c-Myc, $K_2 <$ [c-Myc] $< K_5$, the Hill function describing production by c-Myc will be close to zero, but so will the Hill function describing the inhibitory effect of c-Myc, which will ultimately lead to a low rate of production for Bim. In this case, the production of Bim by Chop is also blocked. We chose this form for the production rate of Bim due to the indirect nature of the inhibitory effect caused by c-Myc. In particular, in this limit, c-Myc prevents Bim translocation to the mitochondria. Thus, even if Chop concentration is sufficient to induce Bim expression, it will not accumulate in the mitochondria to play a role in the apoptosis pathway. Finally, at lower concentrations of c-Myc, [c-Myc] $< K_2$, the Hill function describing production by c-Myc will be close to zero, but the Hill function describing inhibition by c-Myc will be close to one, i.e. the inhibitory effect will be negligible. In this case, expression by Chop will dominate the production of Bim and if Chop expression is high enough, the maximal production rate will be achieved.

The next four terms describe binding and dissociation events with the pro-survival Bcl-2 family proteins, and the following six terms describe binding, dissociation, and activation of the pro-apoptosis effector proteins. The last term describes natural protein decay.

4. The equation describing the rate of change of free Bax concentration is:

$$
\begin{aligned}
\frac{d[\text{Bax}]}{dt} &= k_{0,\text{Bax}} \\
&+ k_{\text{Bax}}\left(\frac{\left(\frac{[\text{c-Myc}]}{K_6}\right)^{n_6}+\left(\frac{[\text{Chop}]}{K_{11}}\right)^{n_{11}}+\left(\frac{[\text{c-Myc}]}{K_6}\right)^{n_6}\left(\frac{[\text{Chop}]}{K_{11}}\right)^{n_{11}}}{1+\left(\frac{[\text{c-Myc}]}{K_6}\right)^{n_6}+\left(\frac{[\text{Chop}]}{K_{11}}\right)^{n_{11}}+\left(\frac{[\text{c-Myc}]}{K_6}\right)^{n_6}\left(\frac{[\text{Chop}]}{K_{11}}\right)^{n_{11}}}\right)\left(\frac{1}{1+\left(\frac{[\text{ABT-199}_{\text{total}}]}{K_{13}}\right)^{n_{13}}}\right) \\
&- k_3[\text{Bim}][\text{Bax}] + k_{-3}[\text{Bim:Bax}] - \delta_{\text{Bax}}[\text{Bax}] \qquad \text{(A.21)}
\end{aligned}
$$

The first term is the basal production rate and the second term describes the stimulatory regulatory effect of c-Myc at high concentrations, the stimulatory regulatory effect of Chop, and the inhibitory effect of venetoclax. The next two terms describe binding and dissociation events with Bim, and the last term describes natural protein decay.

5. The equation describing the rate of change of free Bak concentration is:

$$
\begin{aligned}
\frac{d[\text{Bak}]}{dt} &= k_{0,\text{Bak}} + k_{\text{Bak}}\left(\frac{\left(\frac{[\text{c-Myc}]}{K_7}\right)^{n_7}+\left(\frac{[\text{Chop}]}{K_{12}}\right)^{n_{12}}+\left(\frac{[\text{c-Myc}]}{K_7}\right)^{n_7}\left(\frac{[\text{Chop}]}{K_{12}}\right)^{n_{12}}}{1+\left(\frac{[\text{c-Myc}]}{K_7}\right)^{n_7}+\left(\frac{[\text{Chop}]}{K_{12}}\right)^{n_{12}}+\left(\frac{[\text{c-Myc}]}{K_7}\right)^{n_7}\left(\frac{[\text{Chop}]}{K_{12}}\right)^{n_{12}}}\right) \\
&- k_4[\text{Bim}][\text{Bak}] + k_{-4}[\text{Bim:Bak}] - \delta_{\text{Bak}}[\text{Bak}] \qquad \text{(A.22)}
\end{aligned}
$$

The first term is the basal production rate and the second term describes the stimulatory regulatory effect of c-Myc at high concentrations, and the stimulatory regulatory effect of Chop. The next two terms describe binding and dissociation events with Bim, and the last term describes natural protein decay.

6. The equation describing the rate of change of activated Bax concentration is:

$$\frac{d[\text{Bax*}]}{dt} = \kappa_3[\text{Bim:Bax}] - k_5[\text{Mcl-1}][\text{Bax*}] + k_{-5}[\text{Mcl-1:Bax*}] - k_7[\text{Bcl-2}][\text{Bax*}] + k_{-7}[\text{Bcl-2:Bax*}] - \delta_{\text{Bax}}[\text{Bax*}] \tag{A.23}$$

The first term describes the rate of activation by pro-apoptosis activator protein Bim, the next four terms describe binding and dissociation events with pro-survival Bcl-2 family proteins, and the last term describes natural protein decay.

7. The equation describing the time evolution of activated Bak concentration is:

$$\frac{d[\text{Bak*}]}{dt} = \kappa_4[\text{Bim:Bak}] - k_6[\text{Mcl-1}][\text{Bak*}] + k_{-6}[\text{Mcl-1:Bak*}] - k_8[\text{Bcl-2}][\text{Bak*}] + k_{-8}[\text{Bcl-2:Bak*}] - \delta_{\text{Bak}}[\text{Bak*}] \tag{A.24}$$

The first term describes the rate of activation by pro-apoptosis activator protein Bim, the next four terms describe binding and dissociation events with pro-survival Bcl-2 family proteins, and the last term describes natural protein decay.

8. The equation describing the time evolution of (inactive) Caspase-3 concentration is:

$$\frac{d[\text{Casp-3}]}{dt} = k_{0,\text{Casp-3}} - k_9[\text{Bax*}][\text{Casp-3}] - k_{10}[\text{Bak*}][\text{Casp-3}] - \delta_{\text{Casp-3}}[\text{Casp-3}] \tag{A.25}$$

The first term is the production rate of Caspase-3, the next two terms describe the activation of Caspase-3 by the activated pro-apoptosis effector proteins, and the last term describes natural protein decay.

9. The equation describing the time evolution of activated Caspase-3 concentration is:

$$\frac{d[\text{Casp-3*}]}{dt} = k_9[\text{Bax*}][\text{Casp-3}] + k_{10}[\text{Bak*}][\text{Casp-3}] - \delta_{\text{Casp-3}}[\text{Casp-3*}] \tag{A.26}$$

The first two terms describe the activation of Caspase-3 by the activated pro-apoptosis effector proteins, and the last term describes natural protein decay.

10. The equation describing the time evolution of inactive (cleaved) Mcl-1, denoted Mcl-1$^-$, concentration is:

$$\frac{d[\text{Mcl-1}^-]}{dt} = k_{11}[\text{Mcl-1}][\text{Casp-3*}] - \delta_{\text{Mcl-1}}[\text{Mcl-1}^-] \tag{A.27}$$

The first term describes the cleavage of Mcl-1 by active Caspase-3 and the second term describes natural protein decay.

11. The equation describing the time evolution of inactive (cleaved) Bcl-2, denoted Bcl-2$^-$, concentration is:

$$\frac{d[\text{Bcl-2}^-]}{dt} = k_{12}[\text{Bcl-2}][\text{Casp-3*}] - \delta_{\text{Bcl-2}}[\text{Bcl-2}^-] \tag{A.28}$$

The first term describes the cleavage of Bcl-2 by active Caspase-3 and the second term describes natural protein decay.

12. The equations describing the time evolution of the concentration of protein complexes in the

system are given by:

$$\frac{d[\text{Mcl-1:Bim}]}{dt} = k_1[\text{Mcl-1}][\text{Bim}] - k_{-1}[\text{Mcl-1:Bim}] - \delta_{\text{Mcl-1:Bim}}[\text{Mcl-1:Bim}] \tag{A.29}$$

$$\frac{d[\text{Bcl-2:Bim}]}{dt} = k_2[\text{Bcl-2}][\text{Bim}] - k_{-2}[\text{Bcl-2:Bim}] - \delta_{\text{Bcl-2:Bim}}[\text{Bcl-2:Bim}] \tag{A.30}$$

$$\frac{d[\text{Bim:Bax}]}{dt} = k_3[\text{Bim}][\text{Bax}] - k_{-3}[\text{Bim:Bax}] - \kappa_3[\text{Bim:Bax}] - \delta_{\text{Bim:Bax}}[\text{Bim:Bax}] \tag{A.31}$$

$$\frac{d[\text{Bim:Bak}]}{dt} = k_4[\text{Bim}][\text{Bak}] - k_{-4}[\text{Bim:Bak}] - \kappa_4[\text{Bim:Bak}] - \delta_{\text{Bim:Bak}}[\text{Bim:Bak}] \tag{A.32}$$

$$\frac{d[\text{Mcl-1:Bax*}]}{dt} = k_5[\text{Mcl-1}][\text{Bax*}] - k_{-5}[\text{Mcl-1:Bax*}] - \delta_{\text{Mcl-1:Bax*}}[\text{Mcl-1:Bax*}] \tag{A.33}$$

$$\frac{d[\text{Mcl-1:Bak*}]}{dt} = k_6[\text{Mcl-1}][\text{Bak*}] - k_{-6}[\text{Mcl-1:Bak*}] - \delta_{\text{Mcl-1:Bak*}}[\text{Mcl-1:Bak*}] \tag{A.34}$$

$$\frac{d[\text{Bcl-2:Bax*}]}{dt} = k_7[\text{Bcl-2}][\text{Bax*}] - k_{-7}[\text{Bcl-2:Bax*}] - \delta_{\text{Bcl-2:Bax*}}[\text{Bcl-2:Bax*}] \tag{A.35}$$

$$\frac{d[\text{Bcl-2:Bak*}]}{dt} = k_8[\text{Bcl-2}][\text{Bak*}] - k_{-8}[\text{Bcl-2:Bak*}] - \delta_{\text{Bcl-2:Bak*}}[\text{Bcl-2:Bak*}] \tag{A.36}$$

In the above equations, the first term describes complex formation, the second term describes complex dissociation, and the last term describes natural protein decay. In Eqs. (A.22)-(A.23), the third term describes the activation of pro-apoptosis effector proteins.

13. The equation describing the time evolution of the concentration of the protein-drug complex formed by venetoclax and Bcl-2 is given by:

$$\frac{d[\text{Bcl-2:ABT-199}]}{dt} = k_{13}[\text{Bcl-2}][\text{ABT-199}] - k_{-13}[\text{Bcl-2:ABT-199}] - \delta_{\text{ABT-199}}[\text{Bcl-2:ABT-199}]. \tag{A.37}$$

In the above equation, the first term describes the formation of the protein-drug complex, the second term describes the complex dissociation, and the last term describes natural decay, which is assumed to occur at the same rate as the free intracellular venetoclax concentration, [ABT-199]. The total intracellular drug concentration is thus given by $[\text{ABT-199}_{\text{total}}] = [\text{ABT-199}] + [\text{Bcl-2:ABT-199}]$.

We point out that each combined ODE contains a constant decay rate. This decay is assumed to be the result of natural spontaneous degradation as well as dilution in concentration caused by cell growth [2]. We also note that since each protein in the apoptosis pathway exists in several forms, to obtain the total concentration of each protein, we must sum up the concentration for all of its species. For example, the time-dependent total concentration of Mcl-1 is given by: $[\text{Mcl-1}_{\text{total}}] = [\text{Mcl-1}] + [\text{Mcl-1:Bim}] + [\text{Mcl-1:Bax*}] + [\text{Mcl-1:Bak*}] + [\text{Mcl-1}^{-}]$.

Finally, we note that the full system of coupled ODEs that describes the time evolution of the coupled intrinsic apoptosis-ISR pathway, Figure 2, is given by Eqs. (A.9)–(A.28), along with the equations for cellular drug uptake and the regulation of transcription factors described in Section A.1, and the equations for cellular proliferation and viability described in Section A.6.

B Kinetic parameter values

The values of the kinetic parameters for the cellular drug uptake model and the regulation of the transcription factors c-Myc and Chop, see Section A.1, were previously determined for the MOLM-13 R2 AML cell line [1] and are presented in Table D.

Table D: Nominal kinetic parameter set for the mean-field model with temporal delays in a subset of the drug effects, obtained by fitting to the experimental data for c-Myc and Chop [1]. The sampling ranges for global sensitivity analysis are also presented in the table.

Parameter	Description	Nominal value	Range	Units

$\delta_{T_E,1}$	Extra-cellular decay rate of venetoclax	2.3×10^{-3}	$\ln(2)/240 - \ln(2)/24$	hour^{-1}
$\delta_{T_i,1}$	Intra-cellular decay rate of venetoclax	9.7×10^{-2}	$\ln(2)/240 - \ln(2)/24$	hour^{-1}
$\delta_{T_E,2}$	Extra-cellular decay rate of Tedizolid	8.9×10^{-3}	$\ln(2)/240 - \ln(2)/24$	hour^{-1}
$\delta_{T_i,2}$	Intra-cellular decay rate of Tedizolid	8.1×10^{-2}	$\ln(2)/240 - \ln(2)/24$	hour^{-1}
$k_{T_1,1}$	Factor of increase for maximum production rate of c-Myc	11.43	$0.1 - 100$	–
$\delta_{T_1,0}$	Decay rate of c-Myc in the untreated system	3.851×10^{-1}	$\ln(2)/3 - 3\ln(2)$	hour^{-1}
$K_{1,1,1}$	Half-saturation constant for c-Myc, venetoclax treatment, and drug effect 1 (increased production)	2.147	$0.01 - 5$	[venetoclax]$_0$
$n_{1,1,1}$	Hill coefficient for c-Myc, venetoclax treatment, and drug effect 1	3.284	$0.1 - 10$	–
$K_{1,1,4}$	Half-saturation constant for c-Myc, venetoclax treatment, and drug effect 4 (decreased stability)	3.703	$0.01 - 5$	[venetoclax]$_0$
$n_{1,1,4}$	Hill coefficient for c-Myc, venetoclax treatment, and drug effect 4	1.683	$0.1 - 10$	–
$K_{1,2,1}$	Half-saturation constant for c-Myc, tedizolid treatment, and drug effect 1	4.849	$0.01 - 5$	[tedizolid]$_0$
$n_{1,2,1}$	Hill coefficient for c-Myc, tedizolid treatment, and drug effect 1	2.670	$0.1 - 10$	–
$K_{1,2,4}$	Half-saturation constant for c-Myc, tedizolid treatment, and drug effect 4 (decreased stability)	1.568	$0.01 - 5$	[tedizolid]$_0$
$n_{1,2,4}$	Hill coefficient for c-Myc, tedizolid treatment, and drug effect 4	6.944	$0.1 - 10$	–
$k_{T_2,1}$	Factor of increase for maximum production rate of Chop	382.49	$0 - 1000$	–
$\delta_{T_2,0}$	Decay rate of Chop in the untreated system	1.756×10^{-1}	$\ln(2)/4 - 2\ln(2)$	hour^{-1}
$K_{2,1,1}$	Half-saturation constant for Chop, venetoclax treatment, and drug effect 1 (increased production)	1.288	$0.01 - 5$	[venetoclax]$_0$
$n_{2,1,1}$	Hill coefficient for Chop, venetoclax treatment, and drug effect 1	20.23	$0.1 - 10$	–
$K_{2,1,4}$	Half-saturation constant for Chop, venetoclax treatment, and drug effect 4 (decreased stability)	5.735	$0.01 - 5$	[venetoclax]$_0$
$n_{2,1,4}$	Hill coefficient for Chop, venetoclax treatment, and drug effect 4	0.9208	$0.1 - 10$	–
$K_{2,2,1}$	Half-saturation constant for Chop, tedizolid treatment, and drug effect 1 (increased production)	1.556	$0.01 - 5$	[tedizolid]$_0$
$n_{2,2,1}$	Hill coefficient for Chop, tedizolid treatment, and drug effect 1	10.74	$0.1 - 10$	–
$K_{2,2,4}$	Half-saturation constant for Chop, tedizolid treatment, and drug effect 4 (decreased stability)	8.772	$0.01 - 5$	[tedizolid]$_0$
$n_{2,2,4}$	Hill coefficient for Chop, tedizolid treatment, and drug effect 4	0.6346	$0.1 - 10$	–
$w_{1,1,1}$	Combination treatment weight for c-Myc, venetoclax treatment, and drug effect 1 (increased production)	1.039×10^{-4}	$0 - 5$	–
$w_{1,2,1}$	Combination treatment weight for c-Myc, tedizolid treatment, and drug effect 1 (increased production)	2.423	$0 - 5$	–
$q_{1,4}$	Combination treatment cooperativity for c-Myc, drug effect 4 (decreased stability)	208.90	$0 - 1000$	–
$q_{2,1}$	Combination treatment cooperativity for Chop, drug effect 1 (increased production)	113.76	$0 - 1000$	–
$\tau_{1,1,1}$	Time delay for c-Myc, venetoclax treatment, and drug effect 1 (increased production)	10.36	$0 - 96$	hour
$\tau_{1,2,1}$	Time delay for c-Myc, tedizolid treatment, and drug effect 1 (increased production)	27.42	$0 - 96$	hour

$\tau_{2,2,1}$	Time delay for Chop, tedizolid treatment, and drug effect 1 (increased production)	5.028	$0-96$	hour
$\tau_{2,2,4}$	Time delay for Chop, tedizolid treatment, and drug effect 4 (decreased stability)	6.00	$0-96$	hour

Several of the remaining kinetic parameter values were determined directly from the literature. In Table E, we present the values of the free protein decay rates and protein complex decay rates that were obtained this way.

Table E: Decays rates for the apoptosis proteins and protein complexes, measured for various cell lines and experimental conditions, determined from the literature.

**Parameter**	**Description**	**Value**	**Units**	**Reference**
$\delta_{\text{Mcl-1}}$	Decay rate for Mcl-1 protein	$\frac{\ln(2)}{45}$	min^{-1}	[81–83]
$\delta_{\text{Bcl-2}}$	Decay rate for Bcl-2 protein	$\frac{\ln(2)}{300}$	min^{-1}	[83, 84]
δ_{Bim}	Decay rate for Bim protein	$\frac{\ln(2)}{240}$	min^{-1}	[83, 84]
δ_{Bax}	Decay rate for Bax protein	$\frac{\ln(2)}{24}$	hour^{-1}	[83, 85, 86]
δ_{Bak}	Decay rate for Bak protein	$\frac{\ln(2)}{24}$	hour^{-1}	[83, 85, 86]
$\delta_{\text{Casp-3}}$	Decay rate for Caspase-3 protein	$\frac{\ln(2)}{8}$	hour^{-1}	[87]
$\delta_{\text{Mcl-1:Bim}}$	Decay rate for Mcl-1:Bim protein complex	$\frac{\ln(2)}{150}$	min^{-1}	[83, 88, 89]
$\delta_{\text{Bcl-2:Bim}}$	Decay rate for Bcl-2:Bim protein complex	$\frac{\ln(2)}{75}$	min^{-1}	[83, 89, 90]
$\delta_{\text{Bim:Bax}}$	Decay rate for Bim:Bax protein complex	$\frac{\ln(2)}{24}$	hour^{-1}	[83, 89]
$\delta_{\text{Bim:Bak}}$	Decay rate for Bim:Bak protein complex	$\frac{\ln(2)}{24}$	hour^{-1}	[83, 89]
$\delta_{\text{Mcl-1:Bax*}}$	Decay rate for Mcl-1:Bax* protein complex	$\frac{\ln(2)}{24}$	hour^{-1}	[83, 89]
$\delta_{\text{Mcl-1:Bak*}}$	Decay rate for Mcl-1:Bak* protein complex	$\frac{\ln(2)}{24}$	hour^{-1}	[83, 89]
$\delta_{\text{Bcl-2:Bax*}}$	Decay rate for Bcl-2:Bax* protein complex	$\frac{\ln(2)}{24}$	hour^{-1}	[83, 89]
$\delta_{\text{Bcl-2:Bak*}}$	Decay rate for Bcl-2:Bak* protein complex	$\frac{\ln(2)}{24}$	hour^{-1}	[83, 89]

In Table F, we present the forward and reverse rate constants for the formation of protein complexes in the apoptosis pathway. In addition, we present the corresponding dissociation constants obtained from the literature, as well as the activation rate constants for the pro-apoptosis effector proteins.

Table F: Binding, dissociation, and activation rates for the apoptosis proteins, measured for various cell lines and experimental conditions, determined from the literature. Seconds is abbreviated by "s" and nanomolar concentration is abbreviated by "nM". Following Ref. [83], the dissociation constants K_D and backward constants were obtained from the literature where available and the forward constant was calculated as $k_{\text{forward}} = k_{\text{backward}}/K_D$.

**Parameter**	**Description**	**Value**	**Units**	**Reference**
k_1	Forward rate constant for Mcl-1 and Bim complex formation	1.3×10^{-3}	nM^{-1}s^{-1}	[26]
k_{-1}	Reverse rate constant for Mcl-1 and Bim complex formation	2.6×10^{-4}	s^{-1}	($K_D = 0.2$ nM) [26]
k_2	Forward rate constant for Bcl-2 and Bim complex formation	3.0×10^{-5}	nM^{-1}s^{-1}	[26]
k_{-2}	Reverse rate constant for Bcl-2 and Bim complex formation	1.4×10^{-4}	s^{-1}	($K_D = 4.5$ nM) [26]
k_3	Forward rate constant for Bim and Bax complex formation	2.57×10^{-6}	nM^{-1}s^{-1}	[33, 83, 89]

k_{-3}	Reverse rate constant for Bim and Bax complex formation	2.57×10^{-4}	s^{-1}	($K_D = 100.0$ nM) [33, 83, 89]
κ_3	Activation rate constant for Bax	1.16×10^{-1}	s^{-1}	[83, 89]
k_4	Forward rate constant for Bim and Bak complex formation	2.57×10^{-6}	$nM^{-1}s^{-1}$	[33, 83, 89]
k_{-4}	Reverse rate constant for Bim and Bak complex formation	2.57×10^{-4}	s^{-1}	($K_D = 100.0$ nM) [33, 83, 89]
κ_4	Activation rate constant for Bak	1.16×10^{-1}	s^{-1}	[83, 89]
k_5	Forward rate constant for Mcl-1 and Bax* complex formation	2.6×10^{-8}	$nM^{-1}s^{-1}$	[34, 83, 89]
k_{-5}	Reverse rate constant for Mcl-1 and Bax* complex formation	2.6×10^{-4}	s^{-1}	($K_D = 10,000$ nM) [34, 83, 89]
k_6	Forward rate constant for Mcl-1 and Bak* complex formation	3.25×10^{-5}	$nM^{-1}s^{-1}$	[35, 36, 83, 89]
k_{-6}	Reverse rate constant for Mcl-1 and Bak* complex formation	2.6×10^{-4}	s^{-1}	($K_D = 8.0$ nM) [35, 36, 83, 89]
k_7	Forward rate constant for Bcl-2 and Bax* complex formation	9.33×10^{-6}	$nM^{-1}s^{-1}$	[37, 83, 89]
k_{-7}	Reverse rate constant for Bcl-2 and Bax* complex formation	1.4×10^{-4}	s^{-1}	($K_D = 15.0$ nM) [37, 83, 89]
k_8	Forward rate constant for Bcl-2 and Bak* complex formation	1.4×10^{-8}	$nM^{-1}s^{-1}$	[35, 83, 89]
k_{-8}	Reverse rate constant for Bcl-2 and Bak* complex formation	1.4×10^{-4}	s^{-1}	($K_D = 10,000$ nM) [35, 83, 89]
k_{13}	Forward rate constant for Bcl-2 and ABT-199 complex formation (estimated from lowest dissociation constant for ABT-199 with Bcl-2 proteins)	3.85×10^{-4}	$nM^{-1}s^{-1}$	[83, 89, 91]
k_{-13}	Reverse rate constant for Bcl-2 and ABT-199 complex formation	1.93×10^{-4}	s^{-1}	($K_D = 0.5$ nM) [83, 89, 91]

The remaining parameters were determined by fitting to the experimental data, where the fitting procedure, as well as the procedure for initializing and performing numerical simulations, is described in Section 2.4 of the manuscript. The nominal parameter set determined this way, along with the corresponding parameter search ranges, are presented in Table G. We approximated the search ranges for the initial levels of Bcl-2 family proteins based on ranges that were observed via quantitative Western blotting for several cancer cell lines and patient tumor samples in Ref. [83]. We used a slightly larger range for Mcl-1 levels than were previously observed since our protein data indicated that the upregulation of Mcl-1 was a significant contributor to venetoclax resistance in the MOLM-13 R2 cell line (it was approximately 40 times higher in the resistant versus parental cell line). We used a large range for the half-saturation constants to account for the possibility that some interactions that have been reported in the literature may not be the dominant regulatory interactions in the system. We note that the kinetic parameter values for the equations describing cellular drug uptake and the regulation of transcription factors, see Section A.1, were reported in Supplemental Table 2 of Ref. [1].

As explained in Section 2.4 of the manuscript, the equilibrium simulations were initialized by first turning off all protein-protein interactions, except for the production and decay terms [92]. Thus the initial values of all protein complexes, active pro-apoptosis effector proteins and Caspase levels, and cleaved pro-survival protein levels were taken to be zero. Consequently, the free protein levels, whose time evolution is described by Eqs. (A.18)–(A.22) and (A.25), were initialized to the total initial protein levels reported in Table G. The transcription factor-dependent production rates were calculated from the background production rate, Hill functions, and decay rates, see Table G, for each protein in the apoptosis pathway by making the quasi-steady state [2] assumption. For

Mcl-1, for example, using Eq. (A.18), this leads to:

$$\begin{aligned} k_{\text{Mcl-1}} &= \left(\delta_{\text{Mcl-1}}[\text{Mcl-1}]_0 - k_{0,\text{Mcl-1}}\right)\left(1 + K_1^{n_1}\right)\left(1 + \frac{1}{K_8^{n8}}\right) \\ &= 94.3 \text{ nM min}^{-1} \end{aligned} \tag{B.1}$$

for the values presented in Table G. For the other Bcl-2 family proteins, we find $k_{\text{Bcl-2}} = 2.52$ nM/min, $k_{\text{Bim}} = 0.02195$ nM/min, $k_{\text{Bax}} = 0.5002$ nM/min, and $k_{\text{Bak}} = 0.8604$ nM/min. For Caspase-3, the production rate was set to balance the decay rate since there was no assumed transcription factor-dependent production of this protein.

Lastly, in Table G, we have reported individual search ranges for each rate that contributes to the total cellular proliferation. However, we note that when searching for these parameter values, it was the total doubling rate in the untreated system (which includes multiplication by Hill functions, see Eqs. (A.14)–(A.15)) that was constrained to fall within these previously reported biologically-relevant ranges [93]. In particular, given the value of the initial protein levels, half-saturation constants, Hill coefficients, and proliferation and death rates in Table G, the contribution to cellular proliferation from Bcl-2 is at most 1.09×10^{-3} hour^{-1} and from c-Myc it is 3.39×10^{-5} hour^{-1} in the untreated system. This leads to a maximum rate of cellular proliferation in the system of $4.5 \times 10^{-2} \approx \ln(2)/15.3$ hour^{-1}, i.e. a cell division time of about 15.3 hours, which corresponds to twice the minimum doubling time observed previously in acute leukemia cells [93]. Moreover, to achieve the level of active Caspase-3 that was observed experimentally in the treated system, we imposed a maximum of 0.15 for the active Caspase-3 fraction in the untreated system, leading to an upper limit on the death rate in untreated cells of $7.62 \times 10^{-3} \approx \ln(2)/91.0$ hour^{-1}. Taking into account cellular proliferation and cell death, this corresponds to a doubling time of approximately 18.4 hour^{-1} in the untreated system, which agrees with the experimental data used in this study and falls well within previously reported biologically-relevant ranges.

Table G: Nominal values and search/sampling ranges of the initial protein levels and model kinetic parameters. Nominal values were determined by fitting to the experimental data. Ranges for steady-state protein concentrations were estimated from HeLa cells and colorectal cancer cell lines and patient samples [83]. $[\text{c-Myc}]_0$ and $[\text{Chop}]_0$ refer to the untreated steady-state concentrations of c-Myc and Chop, respectively. $[\text{ABT-199}]_0$ refers to the administered dose of venetoclax treatment and $[\text{Ted}]_0$ refers to the administered dose of tedizolid treatment. See text for further details about cellular proliferation and death parameters.

**Parameter**	**Description**	**Nominal value**	**Range**	**Units**	**Reference**
k_9	Rate of Caspase-3 activation due to Bax*	1.90×10^{-2}	$1 \times 10^{-4} - 1$	nM^{-1}min^{-1}	
k_{10}	Rate of Caspase-3 activation due to Bak* (assumed equal to k_9)	1.90×10^{-2}	$1 \times 10^{-4} - 1$	nM^{-1}min^{-1}	
k_{11}	Rate of Mcl-1 cleavage due to Casp-3*	0.675	$1 \times 10^{-4} - 1$	nM^{-1}min^{-1}	
k_{12}	Rate of Bcl-2 cleavage due to Casp-3* (assumed equal to k_{11})	0.675	$1 \times 10^{-4} - 1$	nM^{-1}min^{-1}	
$[\text{Mcl-1}]_0$	Steady-state untreated total Mcl-1 concentration	1044.1	100 – 2500	nM	[83, 89]
$[\text{Bcl-2}]_0$	Steady-state untreated total Bcl-2 concentration	1153.2	100 – 2500	nM	[83, 89]
$[\text{Bim}]_0$	Steady-state untreated total Bim concentration	7.19	0.5 – 100	nM	
$[\text{Bax}]_0$	Steady-state untreated total Bax concentration	82.7	1 – 1500	nM	[83, 89]

$[\mathrm{Bak}]_0$	Steady-state untreated total Bak concentration	452.2	10 – 3000	nM	[83, 89]
$[\mathrm{Casp\text{-}3}]_0$	Steady-state untreated total Casp-3 concentration	0.162	0.01 – 10	nM	
$k_{0,\mathrm{Mcl\text{-}1}}$	Background production rate for Mcl-1 protein	5.29	$0 - \delta_{\mathrm{Mcl\text{-}1}}[\mathrm{Mcl\text{-}1}]_0 = 0 - 38.5$	nM min^{-1}	Steady-state
$k_{0,\mathrm{Bcl\text{-}2}}$	Background production rate for Bcl-2 protein	0.180	$0 - \delta_{\mathrm{Bcl\text{-}2}}[\mathrm{Bcl\text{-}2}]_0 = 0 - 5.78$	nM min^{-1}	Steady-state
$k_{0,\mathrm{Bim}}$	Background production rate for Bim protein	3.22×10^{-3}	$0 - \delta_{\mathrm{Bim}}[\mathrm{Bim}]_0 = 0 - 0.29$	nM min^{-1}	Steady-state
$k_{0,\mathrm{Bax}}$	Background production rate for Bax protein	1.59×10^{-2}	$0 - \delta_{\mathrm{Bax}}[\mathrm{Bax}]_0 = 0 - 0.722$	nM min^{-1}	Steady-state
$k_{0,\mathrm{Bak}}$	Background production rate for Bak protein	1.17×10^{-1}	$0 - \delta_{\mathrm{Bak}}[\mathrm{Bak}]_0 = 0 - 1.44$	nM min^{-1}	Steady-state
K_1	Half-saturation constant for c-Myc upregulation of Mcl-1	1.73	0.01 – 10	$[\mathrm{c\text{-}Myc}]_0$	
n_1	Hill coefficient for c-Myc upregulation of Mcl-1	3.44	0.5 – 10	–	
K_2	Half-saturation constant for c-Myc upregulation of Bcl-2	0.0148	0.01 – 10 constrained to $< K_4$	$[\mathrm{c\text{-}Myc}]_0$	
n_2	Hill coefficient for c-Myc upregulation of Bcl-2	1.02	0.5 – 10	–	
K_3	Half-saturation constant for c-Myc inhibition of Bim	0.141	0.01 – 10 constrained to $< K_5$	$[\mathrm{c\text{-}Myc}]_0$	
n_3	Hill coefficient for c-Myc inhibition of Bim	1.70	0.5 – 10	–	
K_4	Half-saturation constant for c-Myc inhibition of Bcl-2	3.15	0.5 – 10	$[\mathrm{c\text{-}Myc}]_0$	
n_4	Hill coefficient for c-Myc inhibition of Bcl-2	8.08	0.5 – 10	–	
K_5	Half-saturation constant for c-Myc upregulation of Bim	0.602	0.5 – 10	$[\mathrm{c\text{-}Myc}]_0$	
n_5	Hill coefficient for c-Myc upregulation of Bim	2.72	0.5 – 10	–	
K_6	Half-saturation constant for c-Myc upregulation of Bax	3.52	0.5 – 10	$[\mathrm{c\text{-}Myc}]_0$	
n_6	Hill coefficient for c-Myc upregulation of Bax	2.38	0.5 – 10	–	
K_7	Half-saturation constant for c-Myc upregulation of Bak	1.44	0.5 – 10	$[\mathrm{c\text{-}Myc}]_0$	
n_7	Hill coefficient for c-Myc upregulation of Bak	6.82	0.5 – 10	–	
K_8	Half-saturation constant for Chop inhibition of Mcl-1	2.00	0.01 – 30	$[\mathrm{Chop}]_0$	
n_8	Hill coefficient for Chop inhibition of Mcl-1	2.73	0.5 – 10	–	
K_9	Half-saturation constant for Chop inhibition of Bcl-2	20.2	0.01 – 30	$[\mathrm{Chop}]_0$	
n_9	Hill coefficient for Chop inhibition of Bcl-2	10.5	0.5 – 10	–	

K_{10}	Half-saturation constant for Chop upregulation of Bim	14.7	0.01 – 30	[Chop]$_0$	
n_{10}	Hill coefficient for Chop upregulation of Bim	3.71	0.5 – 10	–	
K_{11}	Half-saturation constant for Chop upregulation of Bax	25.6	0.01 – 30	[Chop]$_0$	
n_{11}	Hill coefficient for Chop upregulation of Bax	3.92	0.5 – 10	–	
K_{12}	Half-saturation constant for Chop upregulation of Bak	1.95	0.01 – 30	[Chop]$_0$	
n_{12}	Hill coefficient for Chop upregulation of Bak	4.62	0.5 – 10	–	
K_{13}	Half-saturation constant for venetoclax inhibition of Bax	0.735	0.01 – 10	[ABT-199]$_0$	
n_{13}	Hill coefficient for venetoclax inhibition of Bax	2.06	0.5 – 10	–	
λ_0	Background rate of cellular proliferation	0.0442	ln(2)/72 – ln(2)/7 total untreated doubling rate	hour^{-1}	[93] See text
$\lambda_{\text{Bcl-2}}$	Bcl-2 dependent rate of cell proliferation	0.0706	ln(2)/72 – ln(2)/7 including Hill function	hour^{-1}	[93] See text
K_{14}	Half-saturation constant for Bcl-2 dependent rate of cell proliferation	1.86	0.01 – 20	[Bcl-2]$_0$	
n_{14}	Hill coefficient for Bcl-2 rate of cell proliferation	6.70	0.5 – 10	–	
$\lambda_{\text{c-Myc}}$	c-Myc dependent rate of cell proliferation	0.144	ln(2)/72 – ln(2)/7 including Hill function	hour^{-1}	[93] See text
K_{15}	Half-saturation constant for c-Myc dependent rate of cell proliferation	11.0	0.01 – 20	[c-Myc]$_0$	
n_{15}	Hill coefficient for c-Myc rate of cell proliferation	3.48	0.5 – 10	–	
K_{16}	Half-saturation constant for Tedizolid dependent inhibition of cellular proliferation	3.72	0.01 – 20	[Ted]$_0$	
n_{16}	Hill coefficient for Tedizolid inhibition of cell proliferation	1.03	0.5 – 10	–	
$\Omega_{\text{Casp-3}}$	Caspase-3 dependent rate of cell death	0.137	ln(2)/72 – ln(2)/7 including Hill function	hour^{-1}	[93] See text
K_{17}	Half-saturation constant for Casp-3* fraction promoting cell death	0.521	0.01 – 1.0	–	
n_{17}	Hill coefficient for Casp-3* fraction promoting cell death	2.27	0.5 – 10	–	

C Sensitivity analysis

In this work, we performed both local and global sensitivity analyses for the kinetic parameters and initial conditions in the mathematical model. Details of the local sensitivity analysis are presented in Sections 2.5 and 3.2 of the manuscript and the results are depicted in Figure 5 and further

Table H: Relative sensitivities determined by local sensitivity analysis of the kinetic parameters and initial conditions, around the nominal parameter set defined by Tables D and G. The top 10 most sensitive parameters are tabulated for each treatment condition and ordered from most to least sensitive. The relative sensitivity determined by perturbation of +1% is presented in the first column, followed by the relative sensitivity corresponding to a −1% perturbation to the parameter value in the second column, for each treatment condition. Parameters that were also identified as significant by global sensitivity analysis are in boldface (c.f. Figure A).

**Untreated**			**Venetoclax**			**Tedizolid**			**Combination**		
**Parameter**	**+1%**	**-1%**	**Parameter**	**+1%**	**-1%**	**Parameter**	**+1%**	**-1%**	**Parameter**	**+1%**	**-1%**
$\mathbf{[Mcl\text{-}1]_0}$	0.1146	−0.1318	$\mathbf{K_{2,1,1}}$	0.2529	−0.5506	$\mathbf{K_{2,2,1}}$	0.4793	−0.7645	$\mathbf{K_{2,1,1}}$	50.17	−35.29
$[Bim]_0$	−0.0830	0.0768	$[Mcl\text{-}1]_0$	0.1245	−0.1462	$\mathbf{n_{2,2,1}}$	0.2510	−0.3112	$\mathbf{K_{2,2,1}}$	28.77	−22.54
$\mathbf{k_9}$	−0.0829	0.0767	$K_{1,1,1}$	−0.1092	0.0998	$[Mcl\text{-}1]_0$	0.2504	−0.2936	$\mathbf{n_{2,2,1}}$	12.90	−11.50
$\mathbf{n_{17}}$	0.0690	−0.0728	k_9	−0.0928	0.0846	k_9	−0.1918	0.1748	$\mathbf{n_{2,1,1}}$	11.94	−10.87
$[Casp\text{-}3]_0$	-0.0565	0.0522	$[Bim]_0$	−0.0927	0.0845	$[Bim]_0$	-0.1914	0.1744	$[Casp\text{-}3]_0$	-11.02	12.05
k_{11}	-0.0565	0.0522	$n_{1,1,1}$	−0.0905	0.0835	$[Casp\text{-}3]_0$	-0.1452	0.1322	k_{11}	-11.018	12.05
$[Bak]_0$	-0.0466	0.0448	$\mathbf{n_{2,1,1}}$	0.0863	-0.1057	k_{11}	-0.1452	0.1322	k_9	-10.77	11.87
$\mathbf{[Bax]_0}$	-0.0347	0.0334	$[Casp\text{-}3]_0$	-0.0687	0.0625	$\mathbf{K_{1,2,4}}$	0.1225	-0.1388	$[Mcl\text{-}1]_0$	9.779	-9.3608
$[Bcl\text{-}2]_0$	0.0326	-0.0344	k_{11}	-0.0687	0.0625	n_{17}	0.1187	-0.1247	$[Bim]_0$	-9.357	10.14
$\mathbf{K_{17}}$	0.0289	-0.0299	$n_{1,1,4}$	0.06779	-0.07369	$\mathbf{[Bak]_0}$	-0.1141	0.1086	$k_{0,\mathrm{Mcl-1}}$	9.312	-8.678

tabulated here in Table H. Following our previous work [94,95], here we used the Latin hypercube sampling (LHS) method [96] to efficiently sample the parameter space (i.e. to generate the parameter sets and initial conditions) for the global sensitivity analysis. The sampling ranges are specified in Table D for the kinetic parameters related to drug uptake and the regulation of the transcription factors c-Myc and Chop and in Table G for the kinetic parameters related to the intrinsic apoptosis pathway. Using the LHS method, we generated 100,000 sets of parameters/initial conditions and simulated the treatment conditions described in Section 2.2 of the manuscript (untreated, venetoclax monotherapy, tedizolid monotherapy, venetoclax/tedizolid combination treatment) for each set. We used multi-parametric sensitivity analysis (MPSA) [97–99], which evaluates the parameter (and initial condition) sensitivities based on Kolmogorov-Smirnov statistics, returning sensitivity values between 0 and 1. A larger parameter sensitivity indicates that the corresponding parameter variation has a large impact on the model output [100]. The results are presented in Figure A for each treatment condition, with the model output taken to be the cell viability at the end of treatment. Inspection of Figure A reveals that the results of the global sensitivity analysis support the results of the local sensitivity analysis results. In particular, kinetic parameters related to the expression of Chop have a significant influence on the response to treatment.

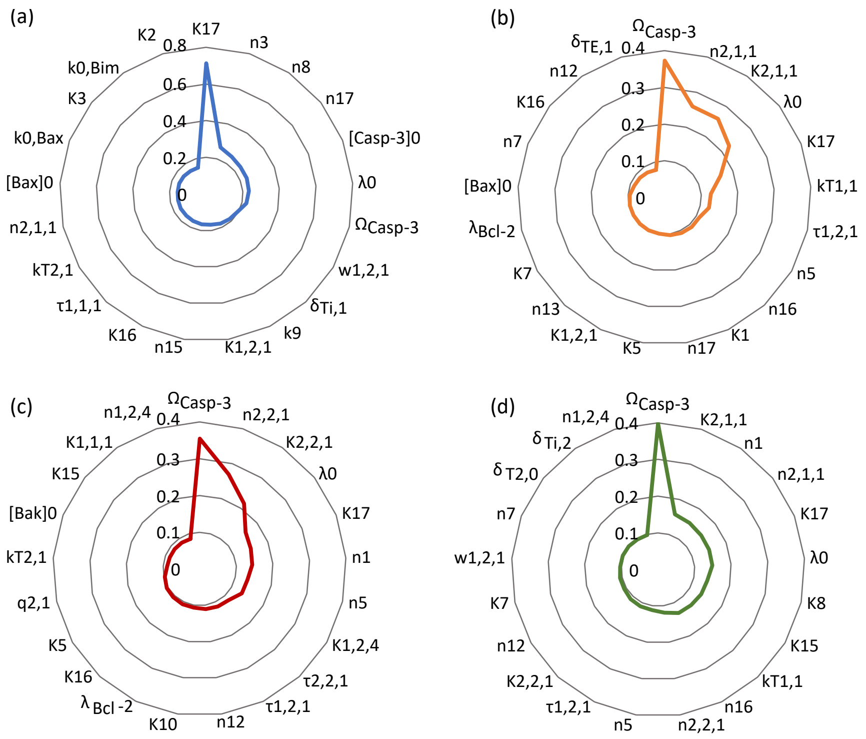

Figure A: MPSA sensitivities determined by global sensitivity analysis for the model kinetic parameters and initial conditions in (a) the untreated system, and during (b) venetoclax monotherapy, (c) tedizolid monotherapy, and (d) venetoclax/tedizolid combination therapy.

References

[1] Michelle Przedborski, David Sharon, Steven Chan, and Mohammad Kohandel. A mean-field approach for modeling the propagation of perturbations in biochemical reaction networks. *European Journal of Pharmaceutical Sciences*, 165:105919, 2021.

[2] Brian P Ingalls. *Mathematical modeling in systems biology: an introduction.* MIT press, 2013.

[3] Archibald Vivian Hill. The possible effects of the aggregation of the molecules of haemoglobin on its dissociation curves. *j. physiol.*, 40:4–7, 1910.

[4] Moises Santillán. On the use of the hill functions in mathematical models of gene regulatory networks. *Mathematical Modelling of Natural Phenomena*, 3(2):85–97, 2008.

[5] Uri Alon. *An introduction to systems biology: design principles of biological circuits.* Chapman and Hall/CRC, 2006.

[6] Wajana L Labisso, Matthias Wirth, Natasa Stojanovic, Roland H Stauber, Angelika Schnieke, Roland M Schmid, Oliver H Krämer, Dieter Saur, and Günter Schneider. Myc directs transcription of mcl1 and eif4e genes to control sensitivity of gastric cancer cells toward hdac inhibitors. *Cell cycle*, 11(8):1593–1602, 2012.

[7] Xiao-Yong Zhang, Harla K Pfeiffer, Hestia S Mellert, Timothy J Stanek, Robyn T Sussman, Alpana Kumari, Duonan Yu, Isidore Rigoutsos, Andrei Thomas-Tikhonenko, Hans E Seidel, et al. Inhibition of the single downstream target bag1 activates the latent apoptotic potential of myc. *Molecular and cellular biology*, 31(24):5037–5045, 2011.

[8] Steven B McMahon. Myc and the control of apoptosis. *Cold Spring Harbor perspectives in medicine*, 4(7):a014407, 2014.

[9] Chi V Dang. c-myc target genes involved in cell growth, apoptosis, and metabolism. *Molecular and cellular biology*, 19(1):1–11, 1999.

[10] Tong Tong, Junfang Ji, Shunqian Jin, Xianxing Li, Wenhong Fan, Yongmei Song, Minrong Wang, Zhihua Liu, Min Wu, and Qimin Zhan. Gadd45a expression induces bim dissociation from the cytoskeleton and translocation to mitochondria. *Molecular and cellular biology*, 25(11):4488–4500, 2005.

[11] B Hoffman and DA Liebermann. Apoptotic signaling by c-myc. *Oncogene*, 27(50):6462–6472, 2008.

[12] Christine M Eischen, David Woo, Martine F Roussel, and John L Cleveland. Apoptosis triggered by myc-induced suppression of bcl-xl or bcl-2 is bypassed during lymphomagenesis. *Molecular and cellular biology*, 21(15):5063–5070, 2001.

[13] Christine M Eischen, Graham Packham, John Nip, Brian E Fee, Scott W Hiebert, Gerard P Zambetti, and John L Cleveland. Bcl-2 is an apoptotic target suppressed by both c-myc and e2f-1. *Oncogene*, 20(48):6983–6993, 2001.

[14] CM Adams and CM Eischen. Histone deacetylase inhibition reveals a tumor-suppressive function of myc-regulated mirna in breast and lung carcinoma. *Cell Death & Differentiation*, 23(8):1312–1321, 2016.

[15] Clare M Adams, Scott W Hiebert, and Christine M Eischen. Myc induces mirna-mediated apoptosis in response to hdac inhibition in hematologic malignancies. *Cancer research*, 76(3):736–748, 2016.

[16] Clare M Adams, Annette S Kim, Ramkrishna Mitra, John K Choi, Jerald Z Gong, Christine M Eischen, et al. Bcl-w has a fundamental role in b cell survival and lymphomagenesis. *The Journal of clinical investigation*, 127(2):635–650, 2017.

[17] Alaa Refaat, Ahmed Abd-Rabou, and Asmaa Reda. Trail combinations: The new 'trail'for cancer therapy. *Oncology letters*, 7(5):1327–1332, 2014.

[18] Ira Tabas and David Ron. Integrating the mechanisms of apoptosis induced by endoplasmic reticulum stress. *Nature cell biology*, 13(3):184, 2011.

[19] Prashanth KB Nagesh, Elham Hatami, Pallabita Chowdhury, Vivek K Kashyap, Sheema Khan, Bilal B Hafeez, Subhash C Chauhan, Meena Jaggi, and Murali M Yallapu. Tannic acid induces endoplasmic reticulum stress-mediated apoptosis in prostate cancer. *Cancers*, 10(3):68, 2018.

[20] Hai Hu, Mingxing Tian, Chan Ding, and Shengqing Yu. The c/ebp homologous protein (chop) transcription factor functions in endoplasmic reticulum stress-induced apoptosis and microbial infection. *Frontiers in immunology*, 9, 2018.

[21] Baltazar D Aguda, Yangjin Kim, Hong Sug Kim, Avner Friedman, and Howard A Fine. Qualitative network modeling of the myc-p53 control system of cell proliferation and differentiation. *Biophysical journal*, 101(9):2082–2091, 2011.

[22] Hyung Don Ryoo and Deepika Vasudevan. Two distinct nodes of translational inhibition in the integrated stress response. *BMB reports*, 50(11):539, 2017.

[23] Yi-Jiun Chen, Bertrand Chin-Ming Tan, Ya-Yun Cheng, Jin-Shin Chen, and Sheng-Chung Lee. Differential regulation of chop translation by phosphorylated eif4e under stress conditions. *Nucleic acids research*, 38(3):764–777, 2009.

[24] Cristina Barbosa, Isabel Peixeiro, and Luísa Romão. Gene expression regulation by upstream open reading frames and human disease. *PLoS genetics*, 9(8):e1003529, 2013.

[25] T Zhou, G Li, B Cao, L Liu, Q Cheng, H Kong, C Shan, X Huang, J Chen, and N Gao. Downregulation of mcl-1 through inhibition of translation contributes to benzyl isothiocyanate-induced cell cycle arrest and apoptosis in human leukemia cells. *Cell death & disease*, 4(2):e515, 2013.

[26] Lin Chen, Simon N Willis, Andrew Wei, Brian J Smith, Jamie I Fletcher, Mark G Hinds, Peter M Colman, Catherine L Day, Jerry M Adams, and David CS Huang. Differential targeting of prosurvival bcl-2 proteins by their bh3-only ligands allows complementary apoptotic function. *Molecular cell*, 17(3):393–403, 2005.

[27] Andreas Strasser, Suzanne Cory, and Jerry M Adams. Deciphering the rules of programmed cell death to improve therapy of cancer and other diseases. *The EMBO journal*, 30(18):3667–3683, 2011.

[28] Wenna Kong, Mi Zhou, Qing Li, Wenjie Fan, Haixia Lin, and Renxiao Wang. Experimental characterization of the binding affinities between proapoptotic bh3 peptides and antiapoptotic bcl-2 proteins. *ChemMedChem*, 13(17):1763–1770, 2018.

[29] Justin Kale, Elizabeth J Osterlund, and David W Andrews. Bcl-2 family proteins: changing partners in the dance towards death. *Cell death and differentiation*, 25(1):65, 2018.

[30] Jerry M Adams and Suzanne Cory. The bcl-2 arbiters of apoptosis and their growing role as cancer targets. *Cell death and differentiation*, 25(1):27, 2018.

[31] Hyungjin Kim, Ho-Chou Tu, Decheng Ren, Osamu Takeuchi, John R Jeffers, Gerard P Zambetti, James J-D Hsieh, and Emily H-Y Cheng. Stepwise activation of bax and bak by tbid, bim, and puma initiates mitochondrial apoptosis. *Molecular cell*, 36(3):487–499, 2009.

[32] Rana Elkholi, Konstantinos V Floros, and Jerry E Chipuk. The role of bh3-only proteins in tumor cell development, signaling, and treatment. *Genes & cancer*, 2(5):523–537, 2011.

[33] Haiming Dai, Alyson Smith, X Wei Meng, Paula A Schneider, Yuan-Ping Pang, and Scott H Kaufmann. Transient binding of an activator bh3 domain to the bak bh3-binding groove initiates bak oligomerization. *Journal of Cell Biology*, 194(1):39–48, 2011.

[34] Marc Germain, Jocelyn Milburn, and Vincent Duronio. Mcl-1 inhibits bax in the absence of mcl-1/bax interaction. *Journal of Biological Chemistry*, 283(10):6384–6392, 2008.

[35] Simon N Willis, Lin Chen, Grant Dewson, Andrew Wei, Edwina Naik, Jamie I Fletcher, Jerry M Adams, and David CS Huang. Proapoptotic bak is sequestered by mcl-1 and bcl-xl, but not bcl-2, until displaced by bh3-only proteins. *Genes & development*, 19(11):1294–1305, 2005.

[36] Haiming Dai, X Wei Meng, Sun-Hee Lee, Paula A Schneider, and Scott H Kaufmann. Context-dependent bcl-2/bak interactions regulate lymphoid cell apoptosis. *Journal of Biological Chemistry*, 284(27):18311–18322, 2009.

[37] Bonsu Ku, Chengyu Liang, Jae U Jung, and Byung-Ha Oh. Evidence that inhibition of bax activation by bcl-2 involves its tight and preferential interaction with the bh3 domain of bax. *Cell research*, 21(4):627–641, 2011.

[38] SJ Korsmeyer, MC Wei, MT Saito, S Weiler, KJ Oh, and PH Schlesinger. Pro-apoptotic cascade activates bid, which oligomerizes bak or bax into pores that result in the release of cytochrome c. *Cell death and differentiation*, 7(12):1166, 2000.

[39] C Garrido, L Galluzzi, M Brunet, PE Puig, C Didelot, and G Kroemer. Mechanisms of cytochrome c release from mitochondria. *Cell death and differentiation*, 13(9):1423, 2006.

[40] Grant Dewson and Ruth M Kluck. Mechanisms by which bak and bax permeabilise mitochondria during apoptosis. *Journal of cell science*, 122(16):2801–2808, 2009.

[41] Stephanie Bleicken, Mirjam Classen, Pulagam VL Padmavathi, Takashi Ishikawa, Kornelius Zeth, Heinz-Jürgen Steinhoff, and Enrica Bordignon. Molecular details of bax activation, oligomerization, and membrane insertion. *Journal of Biological Chemistry*, 285(9):6636–6647, 2010.

[42] Yulia Kushnareva, Alexander Y Andreyev, Tomomi Kuwana, and Donald D Newmeyer. Bax activation initiates the assembly of a multimeric catalyst that facilitates bax pore formation in mitochondrial outer membranes. *PLoS Biol*, 10(9):e1001394, 2012.

[43] Ichiro Nakagawa, Masanobu Nakata, Shigetada Kawabata, and Shigeyuki Hamada. Cytochrome c-mediated caspase-9 activation triggers apoptosis in streptococcus pyogenes-infected epithelial cells. *Cellular microbiology*, 3(6):395–405, 2001.

[44] Xuejun Jiang and Xiaodong Wang. Cytochrome c-mediated apoptosis. *Annual review of biochemistry*, 73, 2004.

[45] Stephen WG Tait and Douglas R Green. Mitochondria and cell death: outer membrane permeabilization and beyond. *Nature reviews Molecular cell biology*, 11(9):621–632, 2010.

[46] Thomas Landes and Jean-Claude Martinou. Mitochondrial outer membrane permeabilization during apoptosis: the role of mitochondrial fission. *Biochimica et Biophysica Acta (BBA)-Molecular Cell Research*, 1813(4):540–545, 2011.

[47] Luke W Thomas, Connie Lam, and Steven W Edwards. Mcl-1; the molecular regulation of protein function. *FEBS letters*, 584(14):2981–2989, 2010.

[48] David G Kirsch, Andrea Doseff, B Nelson Chau, Dae-Sik Lim, Nadja C de Souza-Pinto, Richard Hansford, Michael B Kastan, Yuri A Lazebnik, and J Marie Hardwick. Caspase-3-dependent cleavage of bcl-2 promotes release of cytochrome c. *Journal of Biological Chemistry*, 274(30):21155–21161, 1999.

[49] Lan V Pham, Shengjian Huang, Hui Zhang, Jun Zhang, Taylor Bell, Shouhao Zhou, Elizabeth Pogue, Zhiyong Ding, Laura Lam, Jason Westin, et al. Strategic therapeutic targeting to overcome venetoclax resistance in aggressive b-cell lymphomas. *Clinical Cancer Research*, 24(16):3967–3980, 2018.

[50] Justin Kale, Ozgur Kutuk, Glauber Costa Brito, Tallulah S Andrews, Brian Leber, Anthony Letai, and David W Andrews. Phosphorylation switches bax from promoting to inhibiting apoptosis thereby increasing drug resistance. *EMBO reports*, 19(9):e45235, 2018.

[51] Stephanie Bleicken and Kornelius Zeth. Conformational changes and protein stability of the pro-apoptotic protein bax. *Journal of bioenergetics and biomembranes*, 41(1):29–40, 2009.

[52] Qinhong Wang, Shi-Yong Sun, Fadlo Khuri, Walter J Curran, and Xingming Deng. Mono-or double-site phosphorylation distinctly regulates the proapoptotic function of bax. *PLoS One*, 5(10):e13393, 2010.

[53] Zhi Xiong Chen and Shazib Pervaiz. Bcl-2 induces pro-oxidant state by engaging mitochondrial respiration in tumor cells. *Cell death and differentiation*, 14(9):1617, 2007.

[54] ZX Chen and S Pervaiz. Involvement of cytochrome c oxidase subunits va and vb in the regulation of cancer cell metabolism by bcl-2. *Cell death and differentiation*, 17(3):408, 2010.

[55] Shefali Krishna, Ivan Cherh Chiet Low, and Shazib Pervaiz. Regulation of mitochondrial metabolism: yet another facet in the biology of the oncoprotein bcl-2. *Biochemical Journal*, 435(3):545–551, 2011.

[56] Nathalie Jacque, Anne Marie Ronchetti, Clément Larrue, Godelieve Meunier, Rudy Birsen, Lise Willems, Estelle Saland, Justine Decroocq, Thiago Trovati Maciel, Mireille Lambert, et al. Targeting glutaminolysis has antileukemic activity in acute myeloid leukemia and synergizes with bcl-2 inhibition. *Blood, The Journal of the American Society of Hematology*, 126(11):1346–1356, 2015.

[57] R Bajpai, SM Matulis, C Wei, AK Nooka, HE Von Hollen, S Lonial, LH Boise, and M Shanmugam. Targeting glutamine metabolism in multiple myeloma enhances bim binding to bcl-2 eliciting synthetic lethality to venetoclax. *Oncogene*, 35(30):3955, 2016.

[58] Hong-Duck Um. Bcl-2 family proteins as regulators of cancer cell invasion and metastasis: a review focusing on mitochondrial respiration and reactive oxygen species. *Oncotarget*, 7(5):5193, 2016.

[59] David Sharon, Severine Cathelin, Sara Mirali, Justin M Di Trani, David J Yanofsky, Kristine A Keon, John L Rubinstein, Aaron D Schimmer, Troy Ketela, and Steven M Chan. Inhibition of mitochondrial translation overcomes venetoclax resistance in aml through activation of the integrated stress response. *Science translational medicine*, 11(516), 2019.

[60] Chi V Dang. Myc on the path to cancer. *Cell*, 149(1):22–35, 2012.

[61] Miller Huang and William A Weiss. Neuroblastoma and mycn. *Cold Spring Harbor perspectives in medicine*, 3(10):a014415, 2013.

[62] Martine F Roussel and Giles W Robinson. Role of myc in medulloblastoma. *Cold Spring Harbor perspectives in medicine*, 3(11):a014308, 2013.

[63] Meital Gabay, Yulin Li, and Dean W Felsher. Myc activation is a hallmark of cancer initiation and maintenance. *Cold Spring Harbor perspectives in medicine*, 4(6):a014241, 2014.

[64] Roland Schmitz, Michele Ceribelli, Stefania Pittaluga, George Wright, and Louis M Staudt. Oncogenic mechanisms in burkitt lymphoma. *Cold Spring Harbor perspectives in medicine*, 4(2):a014282, 2014.

[65] Caroline Jose, Nadège Bellance, and Rodrigue Rossignol. Choosing between glycolysis and oxidative phosphorylation: a tumor's dilemma? *Biochimica et Biophysica Acta (BBA)-Bioenergetics*, 1807(6):552–561, 2011.

[66] Alex J Bott, I-Chen Peng, Yongjun Fan, Brandon Faubert, Lu Zhao, Jinyu Li, Sarah Neidler, Yu Sun, Nadia Jaber, Dawid Krokowski, et al. Oncogenic myc induces expression of glutamine synthetase through promoter demethylation. *Cell metabolism*, 22(6):1068–1077, 2015.

[67] Eric S Goetzman and Edward V Prochownik. The role for myc in coordinating glycolysis, oxidative phosphorylation, glutaminolysis, and fatty acid metabolism in normal and neoplastic tissues. *Frontiers in endocrinology*, 9:129, 2018.

[68] Yong Wu, Yu Deng, Jun Zhu, Yachen Duan, WeiWei Weng, and Xiaohua Wu. Pim1 promotes cell proliferation and regulates glycolysis via interaction with myc in ovarian cancer. *OncoTargets and therapy*, 11:6647, 2018.

[69] Yang Dong, Rongfu Tu, Hudan Liu, and Guoliang Qing. Regulation of cancer cell metabolism: oncogenic myc in the driver's seat. *Signal Transduction and Targeted Therapy*, 5(1):1–11, 2020.

[70] Tamara V Milosevic, Valéry L Payen, Pierre Sonveaux, Giulio G Muccioli, Paul M Tulkens, and Françoise Van Bambeke. Mitochondrial alterations (inhibition of mitochondrial protein expression, oxidative metabolism, and ultrastructure) induced by linezolid and tedizolid at clinically relevant concentrations in cultured human hl-60 promyelocytes and thp-1 monocytes. *Antimicrobial agents and chemotherapy*, 62(3):e01599–17, 2018.

[71] Michael J Thomenius and Clark W Distelhorst. Bcl-2 on the endoplasmic reticulum: protecting the mitochondria from a distance. *Journal of Cell Science*, 116(22):4493–4499, 2003.

[72] Qingwen Wan, Ersheng Kuang, Wei Dong, Shumin Zhou, Hua Xu, Yipeng Qi, and Yingle Liu. Reticulon 3 mediates bcl-2 accumulation in mitochondria in response to endoplasmic reticulum stress. *Apoptosis*, 12(2):319–328, 2007.

[73] Stephen WG Tait and Douglas R Green. Mitochondria and cell death: outer membrane permeabilization and beyond. *Nature reviews Molecular cell biology*, 11(9):621, 2010.

[74] Diego Rodriguez, Diego Rojas-Rivera, and Claudio Hetz. Integrating stress signals at the endoplasmic reticulum: the bcl-2 protein family rheostat. *Biochimica et Biophysica Acta (BBA)-Molecular Cell Research*, 1813(4):564–574, 2011.

[75] David R McIlwain, Thorsten Berger, and Tak W Mak. Caspase functions in cell death and disease. *Cold Spring Harbor perspectives in biology*, 5(4):a008656, 2013.

[76] Aisha Shamas-Din, Justin Kale, Brian Leber, and David W Andrews. Mechanisms of action of bcl-2 family proteins. *Cold Spring Harbor perspectives in biology*, 5(4):a008714, 2013.

[77] Rabih Roufayel. Regulation of stressed-induced cell death by the bcl-2 family of apoptotic proteins. *Molecular membrane biology*, 33(6-8):89–99, 2016.

[78] Haidar Akl, Tamara Vervloessem, Santeri Kiviluoto, Mart Bittremieux, Jan B Parys, Humbert De Smedt, and Geert Bultynck. A dual role for the anti-apoptotic bcl-2 protein in cancer: mitochondria versus endoplasmic reticulum. *Biochimica et biophysica acta (BBA)-molecular cell research*, 1843(10):2240–2252, 2014.

[79] Abasha Lewis, Teruo Hayashi, Tsung-Ping Su, and Michael J Betenbaugh. Bcl-2 family in inter-organelle modulation of calcium signaling; roles in bioenergetics and cell survival. *Journal of bioenergetics and biomembranes*, 46(1):1–15, 2014.

[80] Nikolay Popgeorgiev, Lea Jabbour, and Germain Gillet. Subcellular localization and dynamics of the bcl-2 family of proteins. *Frontiers in cell and developmental biology*, 6:13, 2018.

[81] Deepak Nijhawan, Min Fang, Elie Traer, Qing Zhong, Wenhua Gao, Fenghe Du, and Xiaodong Wang. Elimination of mcl-1 is required for the initiation of apoptosis following ultraviolet irradiation. *Genes & development*, 17(12):1475–1486, 2003.

[82] Yoshihisa Kubota, Keiji Kinoshita, Katsutoshi Suetomi, Akira Fujimori, and Sentaro Takahashi. Mcl-1 depletion in apoptosis elicited by ionizing radiation in peritoneal resident macrophages of c3h mice. *The Journal of Immunology*, 178(5):2923–2931, 2007.

[83] Andreas U Lindner, Caoimhín G Concannon, Gerhardt J Boukes, Mary D Cannon, Fabien Llambi, Deborah Ryan, Karen Boland, Joan Kehoe, Deborah A McNamara, Frank Murray, et al. Systems analysis of bcl2 protein family interactions establishes a model to predict responses to chemotherapy. *Cancer research*, 73(2):519–528, 2013.

[84] Stefanie Dimmeler, Kristin Breitschopf, Judith Haendeler, and Andreas M Zeiher. Dephosphorylation targets bcl-2 for ubiquitin-dependent degradation: a link between the apoptosome and the proteasome pathway. *The Journal of experimental medicine*, 189(11):1815–1822, 1999.

[85] Dale A Moulding, Cahit Akgul, Mathieu Derouet, Michael RH White, and Steven W Edwards. Bcl-2 family expression in human neutrophils during delayed and accelerated apoptosis. *Journal of leukocyte biology*, 70(5):783–792, 2001.

[86] Shan Xu, Guihong Peng, Yang Wang, Shengyun Fang, and Mariusz Karbowski. The aaa-atpase p97 is essential for outer mitochondrial membrane protein turnover. *Molecular biology of the cell*, 22(3):291–300, 2011.

[87] John G Walsh, Susan E Logue, Alexander U Lüthi, and Seamus J Martin. Caspase-1 promiscuity is counterbalanced by rapid inactivation of processed enzyme. *Journal of Biological Chemistry*, 286(37):32513–32524, 2011.

[88] Peter E Czabotar, Erinna F Lee, Mark F van Delft, Catherine L Day, Brian J Smith, David CS Huang, W Douglas Fairlie, Mark G Hinds, and Peter M Colman. Structural insights into the degradation of mcl-1 induced by bh3 domains. *Proceedings of the National Academy of Sciences*, 104(15):6217–6222, 2007.

[89] Federico Lucantoni, Andreas U Lindner, Norma O'Donovan, Heiko Düssmann, and Jochen HM Prehn. Systems modeling accurately predicts responses to genotoxic agents and their synergism with bcl-2 inhibitors in triple negative breast cancer cells. *Cell death & disease*, 9(2):42, 2018.

[90] Kristin Breitschopf, Andreas M Zeiher, and Stefanie Dimmeler. Ubiquitin-mediated degradation of the proapoptotic active form of bid: a functional consequence on apoptosis induction. *Journal of Biological Chemistry*, 275(28):21648–21652, 2000.

[91] Guillaume Lessene, Peter E Czabotar, and Peter M Colman. Bcl-2 family antagonists for cancer therapy. *Nature reviews Drug discovery*, 7(12):989–1000, 2008.

[92] Tomas Tokar and Jozef Ulicny. The mathematical model of the bcl-2 family mediated momp regulation can perform a non-trivial pattern recognition. *PloS one*, 8(12):e81861, 2013.

[93] Howard E Skipper and Seymour Perry. Kinetics of normal and leukemic leukocyte populations and relevance to chemotherapy. *Cancer Research*, 30(6):1883–1897, 1970.

[94] Munisha Smalley, Michelle Przedborski, Saravanan Thiyagarajan, Moriah Pellowe, Amit Verma, Nilesh Brijwani, Debika Datta, Misti Jain, Basavaraja U Shanthappa, Vidushi Kapoor, et al. Integrating systems biology and an ex vivo human tumor model elucidates pd-1 blockade response dynamics. *Iscience*, 23(6):101229, 2020.

[95] Michelle Przedborski, Munisha Smalley, Saravanan Thiyagarajan, Aaron Goldman, and Mohammad Kohandel. Systems biology informed neural networks (sbinn) predict response and novel combinations for pd-1 checkpoint blockade. *Communications Biology*, 4(1):1–15, 2021.

[96] Michael D McKay, Richard J Beckman, and William J Conover. A comparison of three methods for selecting values of input variables in the analysis of output from a computer code. *Technometrics*, 42(1):55–61, 2000.

[97] Kwang-Hyun Cho, Sung-Young Shin, Walter Kolch, and Olaf Wolkenhauer. Experimental design in systems biology, based on parameter sensitivity analysis using a monte carlo method: A case study for the tnfα-mediated nf-κ b signal transduction pathway. *Simulation*, 79(12):726–739, 2003.

[98] George M Hornberger and Robert C Spear. Approach to the preliminary analysis of environmental systems. *J. Environ. Mgmt.*, 12(1):7–18, 1981.

[99] Zhike Zi, Kwang-Hyun Cho, Myong-Hee Sung, Xuefeng Xia, Jiashun Zheng, and Zhirong Sun. In silico identification of the key components and steps in ifn-γ induced jak-stat signaling pathway. *FEBS letters*, 579(5):1101–1108, 2005.

[100] Zhike Zi. Sensitivity analysis approaches applied to systems biology models. *IET systems biology*, 5(6):336–346, 2011.
